# Effects of Scarification, Phytohormones, Soil Type, and Warming on the Germination and/or Seedling Performance of Three Tamaulipan Thornscrub Forest Species

**DOI:** 10.3390/plants10081489

**Published:** 2021-07-21

**Authors:** Paula Luera, Kimberly Wahl-Villarreal, Bradley O. Christoffersen, Abeny Treviño, Pushpa Soti, Christopher A. Gabler

**Affiliations:** 1 School of Earth, Environmental, and Marine Sciences, University of Texas Rio Grande Valley, 1 West University Blvd, Brownsville, TX 78520, USA; pluera.luera@austin.utexas.edu (P.L.); bradley.christoffersen@utrgv.edu (B.O.C.); pushpa.soti@utrgv.edu (P.S.); 2 South Texas National Wildlife Refuge Complex, United States Fish and Wildlife Service, 3325 Green Jay Rd, Alamo, TX 78516, USA; kwahl@blm.gov; 3 Department of Biology, University of Texas Rio Grande Valley, 1201 W University Dr, Edinburg, TX 78539, USA; abeny.trevino01@utrgv.edu

**Keywords:** reforestation, germination, propagation, phytohormones, scarification, gibberellic acid, indole-3-butyric acid, Fabaceae, Boraginaceae, Rutaceae

## Abstract

The Tamaulipan thornforests of south Texas and northeast Mexico are an ecologically and economically important conservation hotspot. Thornforest restoration is limited by native tree and shrub seedling availability for planting. Seedling shortages arise from low seed availability and knowledge gaps regarding best practices for germinating and growing the 70+ thornforest species desired for restoration plantings. To fill key knowledge gaps, we investigated three ecologically important thornforest species with low or highly variable germination or seedling survival rates: *Ebenopsis ebano*, *Cordia boissieri*, and *Zanthoxylum fagara*. For each, we quantified the effects of different dosages of chemical seed treatments used to promote germination (sulfuric acid, SA; gibberellic acid, GA; indole-3-butyric acid, IBA) on germination likelihood and timing. We also quantified the effects that these chemical seed treatments, soil media mixture type, and soil warming had on seedling survival, growth, and root morphology. *Ebenopsis* germination peaked (>90%) with 40–60 min SA treatment. *Cordia* germination peaked (40%) with 100 mg/L GA treatment. *Zanthoxylum* germination was negligible across all treatments. Seed molding was rare but stirring during SA treatment reduced *Ebenopsis* molding by 4%. *Ebenopsis* seedling survival, height, leaf count, and root morphology were minimally affected by seed treatments, generally reduced by warming, and influenced by soil mix, which also mediated responses to warming. These results suggest improvements to existing practices that could increase *Ebenopsis* germination by 10–20% and potentially double *Cordia* germination.

## 1. Introduction

Deforestation is both a driver and consequence of climate change, while reforestation offers a means to mitigate climate change. Beyond directly influencing the survival and performance of individual plants, changes in temperature and precipitation regimes alter seasonal cycles and ecological cues that govern population-level reproduction and recruitment, which impact plant species distributions and thus ecosystem functions [1,2,3]. Combined with human population growth, agriculture expansion, urbanization, and other land use changes, climate change exacerbates already extensive habitat loss [4,5,6]. To reverse habitat loss and mitigate climate change while also supporting human populations, many land management techniques aim to conserve or restore habitats that provide multiple important ecological functions, such as wildlife habitat or carbon sequestration [7,8], and often promote forest conservation and restoration in urban and rural sites [9,10].

Tamaulipan (or Mezquital) thornscrub forests (or thornforests) are ecologically and economically valuable habitats that provide an array of important ecosystem functions in the Lower Rio Grande Valley (RGV) region of southernmost Texas and northeastern Mexico. Principal among these functions are wildlife habitat, which supports a hundred-million-dollar regional ecotourism industry based largely around bird- and butterfly-watching, and carbon sequestration [11,12,13]. These dense and well-armed forests provide a short, thick canopy preferred by many native reptiles and mammals, including endangered ocelots (*Leopardus pardalis*) that depend on closed-canopy thornforests with >95% cover [14,15,16]. Thornscrub forests also provide forage and habitat for a particularly high diversity and abundance of bees, beetles, resident and migratory birds and butterflies, and many other organisms [17]. Fruits of many thornscrub plant species are edible to humans (e.g., *Phaulothamnus spinescens*) or have medicinal properties (e.g., *Zanthoxylum fagara*) [18]. Several Tamaulipan thornforest plant species, such as *Ebenopsis ebano* (Texas ebony; Fabaceae) and *Sabal mexicana* (Rio Grande palmetto; Arecaceae), are found nowhere else in the United States [19].

Less than 2% of historic Tamaulipan thornforests remain due to land conversion for human use [20]. Due to its high human land use and high biodiversity, the Tamaulipan ecoregion has been identified as a conservation hotspot [21]. For these and other reasons, restoration of thornscrub forests has become a primary goal of various governmental, conservation, and corporate organizations who operate in the region and have collaborated in producing and planting native thornscrub seedlings. Thornscrub plants are also increasingly being utilized in urban environments to help alleviate habitat fragmentation and better conserve water and soil.

Currently, the supply of native thornscrub plant seedlings does not meet demand (primarily for use in habitat restoration). In fact, thornscrub plant seedling availability is presently considered to be the greatest limiting factor for thornscrub forest restoration [22]. Thornforest restorations predominately utilize woody species and overwhelmingly plant seedlings, rather than seeds, because (a) large-scale plantings (tens of acres/hectares) of seedlings have been more successful in recent decades, and (b) seed availability is also highly limited, and seed predators and parasites are abundant in the region, so nursery production more fully utilizes available seed stocks [22]. In keeping with established best practices, to increase genetic diversity in restored habitats, all seedlings utilized by the United States Fish and Wildlife Service (USFWS) in south Texas must be grown from seed, and other regional actors have similar policies. However, production from seed is relatively labor-intensive, and germination rates are often low and/or inconsistent [22,23].

Low or inconsistent germination rates and, for some species, high seedling mortality rates are at least partly due to a great scarcity of information about the best cultural practices for the more than 72 thornscrub plant species regularly used in habitat restoration projects. Species-specific rearing practices (if established) increase upfront costs for commercial growers. As a result, commercial growers typically follow generalized horticultural methods that are suboptimal, at best, for many species. Altogether, these higher levels of risk (due to propagation knowledge gaps) and higher production costs have discouraged commercial growers from producing thornscrub plant species, despite their high demand. Filling these knowledge gaps should encourage more commercial production and thereby increase both thornscrub seedling availability and habitat restoration.

This study focuses on three woody plant species native to Tamaulipan thornscrub forests: *Ebenopsis ebano* (Berl.) Barneby and Grimes (hereafter *Ebenopsis*), *Cordia boissieri* A. DC. (hereafter *Cordia*), and *Zanthoxylum fagara* (L.) Sarg. (hereafter *Zanthoxylum*). These species were selected based on their ecological importance, the high demand for these species in restoration projects, and because they have either proven difficult to germinate (*Zanthoxylum*), to have highly variable germination rates (*Ebenopsis* and *Cordia*), or to be relatively difficult to rear after germination (*Cordia*) [22].

In wild plant communities, seed viability and germination rates influence plant abundance, but germination patterns and requirements can differ broadly because of variation in ecological strategies and selective pressures [24,25,26]. Specific germination niches have evolved in response to these selective pressures, and seed dormancy is typically the mechanism that prevents a seed from germinating when conditions are likely to reduce the probability of seedling survival [27]. Baskin and Baskin [27] identify five classes of seed dormancy: physical (water-impermeable seed or fruit coats prevent imbibition), physiological (specific environmental conditions alter hormone levels to trigger germination) [24,25,28,29], morphological [30], morphophysiological, and combinational.

This study was most concerned with physical and physiological dormancy because both are known or suspected to be operational in our focal species [22,31], and because both can be synthetically broken. Many general and taxon-specific horticultural techniques exist to break these dormancies [32], but information is scarce on the exact types of dormancy exhibited by Tamaulipan thornscrub species or how thornscrub species respond to horticultural approaches to breaking seed dormancy. These are key knowledge gaps that, once filled, will reduce uncertainty and risk for growers and may help alleviate the limitations of seed availability, if methods to enhance germination rates are identified.

Seeds with water-impermeable seed coats, such as *Ebenopsis*, exhibit physical dormancy and require physical or chemical scarification to trigger germination [27,33]. Abrasion of the seed coat breaks dormancy [25] by permitting gas exchange and imbibition through specialized structures [34] or by creating one or more small channels [35]. Various natural modes of scarification exist, including gut passage and physical weathering [36,37], which can be replicated by growers via mechanical or chemical methods [38]. A common approach is to soak or coat seeds in concentrated sulfuric acid (SA) for a prescribed period of time before neutralizing the acid with agricultural lime [35,39]. This process is often more cost- and labor-efficient than mechanical scarification methods like nicking, piercing, or filing, especially with large quantities of seed. However, sulfuric acid can have detrimental effects on germination for some species [40], and overexposure of seeds to sulfuric acid can kill embryos.

Gibberellic acid (GA or GA3) is a natural phytohormone that promotes cell growth [41,42] and is critical to breaking dormancy and triggering germination [29,32,43]. In seeds, GA induces imbibition and mitotic cell division [28,44] buts its action is countered by abscisic acid (ABA), a phytohormone that promotes dormancy and inhibits germination [28,29,43,45]. Increasing GA concentration in seed tissues can overwhelm ABA inhibition and induce germination or shorten dormancy for some species, especially those with physiological dormancy. GA treatment has become a standard approach in agriculture, horticulture, and plant sciences; however, excess GA can decrease germination [46] and can produce undesirable morphological side effects [47]. For seeds with simple environmental cues, GA treatment is not always economical. For example, many species require only cold or warm stratification or dry after-ripening to break physiological dormancy, and each of these can be cheaper to impose than GA treatment [27,32].

Indole-3-butryic acid (IBA) is also a natural phytohormone (an auxin) that promotes root formation and growth [48,49]. IBA treatment has also become commonplace but is typically used for clonal propagation or to stimulate root growth in transplants. However, recent studies show IBA can also promote germination [50] and may interact with GA in positive or synergistic ways [28]. The effects of IBA on germination of thornscrub species has not been studied.

Soil characteristics are fundamental to plant survival and performance and can have very strong effects in early development. Tamaulipan thornscrub habitats possess soil types ranging from deep clays rich in organic matter in coastal and riparian zones to shallow calcareous sands further inland [51]. We have only a basic understanding of thornscrub seedlings’ soil requirements, and this likely contributes to suboptimal survival and performance of nursery-produced thornscrub seedlings. Optimized soil formulations could improve seedling performance and thus restoration success, while reducing risk and increasing profit for growers, thereby promoting commercial seedling production.

Heating mats are often used to promote seedling growth during cold conditions, and soil warming can have strong positive effects on root growth of many woody species [52,53]. Soil warming also alters the activity of beneficial and pathogenic soil organisms and the overall balance of their interactions with plants [54]. Seedling survival after planting is relatively low among thornscrub reforestation projects in south Texas, with much mortality attributable to drought stress and herbivory [22,55,56]. Seedlings with better-developed root systems should be more resilient to both stressors, so approaches that promote root development could have large impacts on the success of planting efforts.

To better understand the dormancy status and methods required to break dormancy for *Ebenopsis*, *Cordia*, and *Zanthoxylum*, we subjected the seeds of each species to three common horticultural treatments i.e., sulfuric acid, gibberellic acid, and indole-3-butyric acid. For *Ebenopsis*, we also examined the effects of different growing media (soil mixes) and soil warming on the survival and performance of seedlings produced from these seeds. The same experiments are merited for *Cordia* and *Zanthoxylum* seedlings, and would have been performed here, but neither species produced enough seedlings within our germination experiments to permit such studies. The applied purpose was to identify (a) specific seed treatment protocols that maximize the germination of our focal thornscrub species and (b) horticultural best practices that maximize seedling performance of *Ebenopsis*. The information generated by this study fills important knowledge gaps and provides valuable guidelines for the propagation of three ecologically important thornscrub plant species, all of which are in high demand for restoration purposes.

## 2. Results

Table 1 summarizes the results of our chemical seed treatments for all focal species.

### 2.1. Ebenopsis ebano Germination

**Figure 1:**
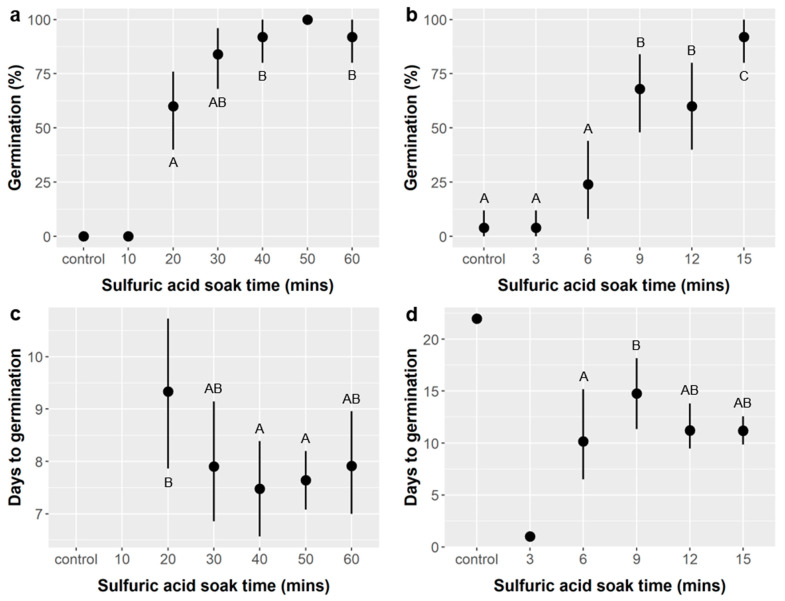
Treatment means with 95% confidence intervals showing the effects of sulfuric acid (SA) soaking time on *Ebenopsis ebano* germination likelihood for (**a**) unstirred and (**b**) stirred seeds, and on the time to germination for (**c**) unstirred and (**d**) stirred seeds. Capital letters denote the results of least square means post-hoc tests; treatments that share a letter were not significantly different. Treatments without capital letters denote cases where post-hoc comparisons were not possible because there was no observed variation (i.e., if germination was 0% or 100% or there was only one observation). Germination likelihood increased as SA soaking time increased, but there were no clear patterns for the time to germination, except that germination was faster in the unstirred treatments.

Sulfuric acid (SA) treatment: Soaking time in sulfuric acid had a significant effect on the likelihood of germination in both the unstirred (ANODEV, χ^2^ = 150.33, *p* < 0.0001) and stirred (ANODEV, χ^2^ = 80.81, *p* < 0.0001) treatments (Figure 1a,b). Germination likelihood was <5% in the control and lowest SA soaking time treatments and increased with soaking time in both the stirred and unstirred treatments. Germination rates of 92–100% were observed with 15 min of soaking in the stirred treatment and with 40, 50, or 60 min of soaking without stirring.

SA soak time also significantly influenced time to germination in the stirred (ANOVA, F_5,57_ = 11.08, *p* < 0.0001) but not the unstirred treatments (ANOVA, F_4,102_, *p* = 0.27) (Figure 1c,d). In the unstirred treatment, the average germination time was 9.3 days with 20 min of soaking and <8 days for all soaking times from 30–60 min, and our post-hoc tests suggest that seeds in the 40 and 50 min treatments did germinate significantly faster (7.5 days) than those in 20 min treatment. In the stirred treatment, idiosyncratic single values were observed for the control and 3 min treatments, and the average times to germination ranged from 10.6 days in the 6 min treatment to 14.8 days in the 9 min treatment. It appears that both germination likelihood and time to germination approached their optima within the range of times tested. Soaking times over 60 min are more likely to damage embryos and reduce germination likelihood and/or slow germination timing, and are not advised given germination of over 90% with 40–60 min.

The likelihood of losing a seed to molding was < 3% overall and independent of SA soaking time in both the stirred (ANODEV, χ^2^ = 3.62, *p* = 0.61) and unstirred treatments (ANODEV, χ^2^ = 9.24, *p* = 0.16), and SA soak time had no effect on the time to molding (ANOVA, F_3,4_ = 1.05, *p* = 0.46) (not shown). We did not have enough observations to analyze time to molding in the stirred treatment, where only two seeds molded.

**Figure 2:**
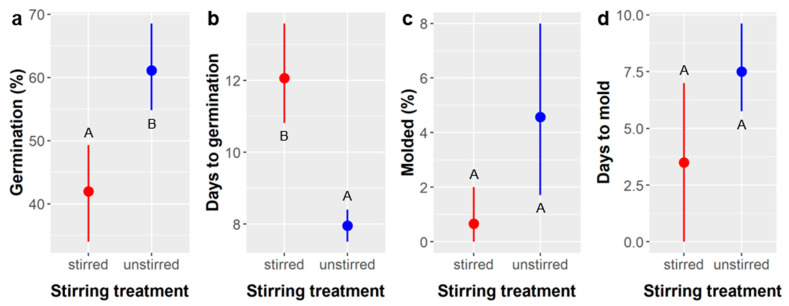
Treatment means with 95% confidence intervals showing the effects of sulfuric acid (SA) stirring treatments on *Ebenopsis ebano* (**a**) germination likelihood (*p* = 0.0006), (**b**) days to germination (*p* < 0.0001), (**c**) likelihood of molding (*p* = 0.0213), and (**d**) days to molding (*p* = 0.17). Capital letters denote the results of least square means post-hoc tests. Germination was significantly more likely and faster in the unstirred treatments, but so was the likelihood of mold. Molding occurred more rapidly in the stirred treatment, but mold observations were scarce, and the difference was not significant.

We could not definitively test the effects of stirring itself in the SA treatments because the stirring treatment was confounded with differences in soaking times (we were likely to kill the seeds if we soaked the stirred seeds much longer). Nevertheless, direct comparisons between the stirring treatments (3–15 min soak time) and unstirred treatments (10–60 min soak time) showed that germination was 19% more likely (ANODEV, χ^2^ = 11.93, *p* = 0.0006) and 4.1 days faster (ANOVA, F_1,168_, *p* < 0.0001) in the unstirred treatment (Figure 2a,b). However, the likelihood of losing seeds to molding was also 4% higher if unstirred (ANODEV, χ^2^ = 5.30, *p* = 0.0213) (Figure 2c). Molding occurred more rapidly in the stirred treatments, but there were few observations of mold and the difference was not significant (ANOVA, F_1,8_, *p* = 0.17) (Figure 2d). Comparisons between the stirred and unstirred treatments with similar soak times suggest stirring was more effective at promoting germination: we saw 68% and 60% germination for the 9 and 12 min stirred treatments, respectively, versus 0% in the 10 min unstirred treatment, and observed 92% germination in the 15 min stirred treatment versus 60% germination with 20 min of unstirred SA soaking. However, these were the shortest unstirred treatments and germination likelihood was highest among all SA soaking treatments in the longer unstirred treatments. These data support the hypothesis that stirring simply speeds the physical degradation of the seed coat during sulfuric acid scarification. However, the differences in time to germination between the stirred and unstirred treatments suggest that, although both appear to effectively breach the seed coat, the resulting permeability is not equal, and imbibition takes longer in shorter stirring treatments compared to longer unstirred treatments. This difference in permeability might result because the shorter stirred treatments create more micropores or small fissures in the seed coat compared to larger gaps created by longer unstirred treatments.

**Figure 3:**
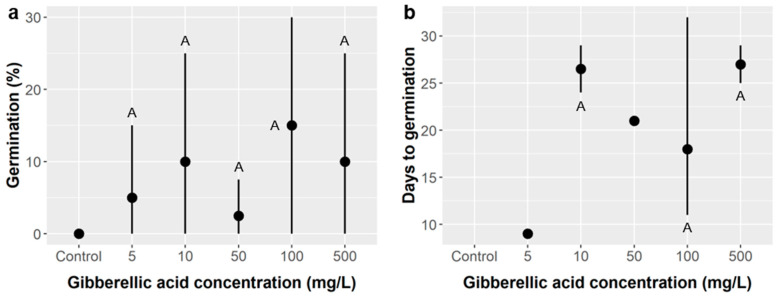
Treatment means with 95% confidence intervals showing the effects of gibberellic acid (GA) treatments on *Ebenopsis ebano* (**a**) germination likelihood (*p* = 0.25) and (**b**) days to germination (*p* = 0.51). Capital letters denote the results of least square means post-hoc tests. Treatments without capital letters denote cases where post-hoc comparisons were not possible because there was no observed variation (i.e., if germination was 0% or 100% or there was only one observation). The likelihood of seed loss due to molding was also independent of GA treatment (*p* = 0.58), but not enough seeds molded to analyze the effect of GA treatment on time to molding.

Gibberellic acid (GA) treatment: Only 6.4% of *Ebenopsis* seeds germinated when treated with gibberellic acid alone. Zero seeds germinated in the control, and germination likelihood ranged from 3% when treated with 5 mg/L GA to 15% with 100 mg/L GA, but these differences were not significant and all treatment levels were indistinguishable from the control (ANODEV, χ^2^ = 6.60, *p* = 0.25) (Figure 3a). Average time to germination ranged from 18 days at 100 mg/L GA to 27 days at 10 and 500 mg/L GA, but these differences were also not significant (ANOVA, F_4,4_ = 0.96, *p* = 0.51) (Figure 3b). Three seeds molded, but GA concentration did not influence the likelihood of molding (ANODEV, χ^2^ = 3.76, *p* = 0.58), and this was not enough observations to analyze the effects of GA concentration on time to molding.

Indole-3-butyric acid (IBA) treatment: Zero *Ebenopsis* seeds germinated in the IBA trial, whether or not they were treated with IBA powder. Several seeds molded, but this was not analyzed because it was uncommon and nothing germinated, which was our focal metric.

### 2.2. Cordia boissieri Germination

Sulfuric acid (SA) treatments: One *Cordia* seed germinated in the 120 min soaking time treatment; it was the only germinant across all seven SA treatments. With one germinant, no analyses were possible. Visual inspection following SA treatments suggested that soaking the *Cordia* seeds in SA for 20–120 min produced the anticipated effect, namely a range of degradation to otherwise intact seed coats. Thus, because we saw equally low germination across all SA treatments, including the control and those with the lowest SA soak times, we do not believe that these extremely low germination rates were the result of over-degradation of the seed coat and damage to embryos by excessive SA exposure.

Gibberellic acid (GA) treatments: Overall *Cordia* germination in the gibberellic acid trial was 24.4% and the average time to germination was 9.8 days. Germination likelihood was significantly influenced by GA concentration, whether the seed coat was heat cracked, and the interaction between GA concentration and heat cracking (Table 2).

**Figure 4:**
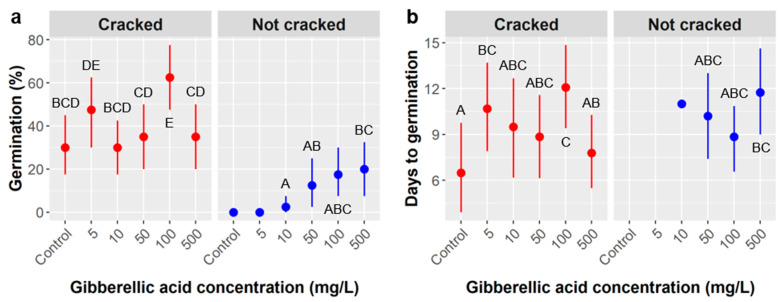
Treatment means with 95% confidence intervals showing the effects of gibberellic acid (GA) and seed coat treatments on (**a**) germination likelihood and (**b**) time to germination of *Cordia boissieri*. Capital letters denote the results of least square means post-hoc tests; treatments that share a letter were not significantly different. Treatments without capital letters denote cases where post-hoc comparisons were not possible because there was no observed variation (i.e., if germination was 0% or 100% or there was only one observation). The main effects of the GA concentration and seed coat treatments were significant, as was the GA × seed coat interaction (Table 2), which is illustrated in panel a and reflects the fact that the relationship between GA concentration and germination was different in the two seed coat treatments. In contrast to the post-hoc tests shown in panel b, the associated ANOVA found that time to germination was not significantly influenced by GA concentration, seed coat treatment, or their interaction (Table 3).

Germination likelihood was 40% in heat cracked seeds but only 9% in uncracked seeds, and there was an overall trend for germination likelihood to increase as GA concentration increased, but this relationship between germination and GA concentration was not consistent between the seed coat cracking treatments (Figure 4a). Among seeds with an uncracked seed coat, there was zero germination in the control and lowest (5 mg/L) GA treatment, and germination likelihood increased gradually as GA concentration increased, reaching 20% in the highest (500 mg/L) GA treatment. However, there was no such trend for seeds with heat cracked coats, whose germination was generally consistent across all GA treatments, except that germination in the 100 mg/L GA treatment was the highest overall at 63% and significantly higher than in all other GA treatments except the 5 mg/L GA treatment, where germination was 48% (Figure 4a). This difference in the relationship between GA concentration and germination likelihood within the two seed coat treatments is the reason for the significant GA × seed coat interaction.

Time to germination ranged from 6.5 days in the control to 11.4 days in the 100 mg/L GA treatment and was 0.7 days faster in seeds with heat cracked seed coats, but these differences were not significant according to our ANOVA, nor was the interaction between GA and seed coat treatments (Table 3, Figure 4b). The associated least square means post-hoc tests did, however, detect significant differences between the cracked control treatment and the three treatments that were slowest to germinate (cracked 5 mg/L, uncracked 500 mg/L, and cracked 100 mg/L). None of the *Cordia* seeds molded in this trial, so we were not able to analyze mold likelihood or time to molding.

Indole-3-butyric acid (IBA) treatment: Zero *Cordia* seeds germinated in the IBA trial, whether or not they were treated with IBA powder. As with *Ebenopsis*, several seeds molded, but this was not analyzed because it was uncommon, and no seeds germinated.

### 2.3. Zanthoxylum fagara Germination

Only one *Zanthoxylum* seed germinated in the sulfuric acid treatments; it was within the unstirred 80-s soak time treatment. Zero *Zanthoxylum* seeds germinated in the gibberellic acid treatments or the indole-3-butyric acid treatments. With only one germinant in the SA treatments and zero germinants in the GA or IBA treatments, we were unable to perform any analyses investigating the effects of SA, GA, or IBA on *Zanthoxylum* germination.

### 2.4. Ebenopsis Seedling Survival and Aboveground Growth

Seedling survival: Overall, 92.6% of *Ebenopsis* seedlings survived until the final data collection in March 2020, which occurred 28–104 days (mean = 66.8 days) after seeds germinated and young seedlings were transplanted into soil mixture treatments and began being reared outdoors. Sulfuric acid treatment had no effect on *Ebenopsis* seedling survival, but the main effects of both soil mixture type and warming via heating mat significantly influenced survival (Table 4).

**Figure 5:**
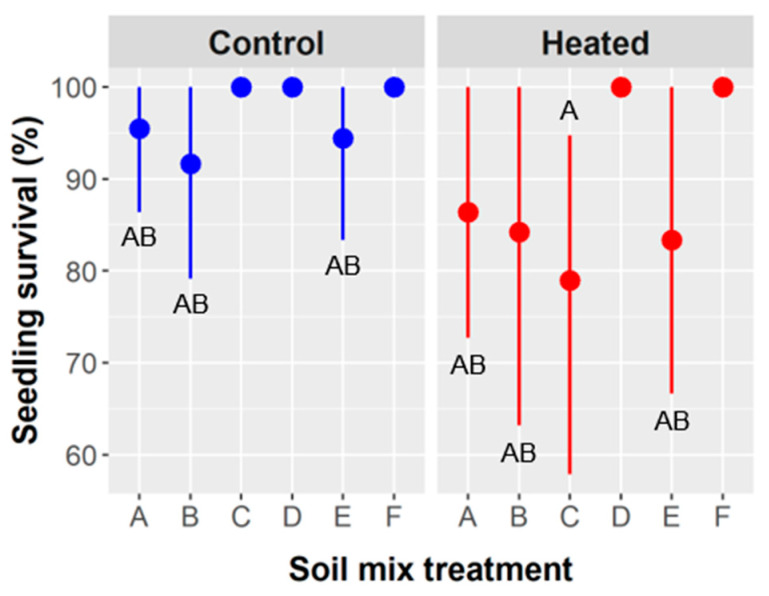
Treatment means with 95% confidence intervals showing the effects of soil mixture treatments on *Ebenopsis ebano* seedling survival, broken down by soil heating treatment. Capital letters denote the results of least square means post-hoc tests. Treatments without capital letters denote cases where post-hoc comparisons were not possible because there was no observed variation (i.e., if survival was 100%). ANODEV results suggests the main effects of soil mixture (*p* = 0.0266) and heating (*p* = 0.0041) influenced survival, but the interaction of soil type and heating illustrated here did not (*p* = 0.70).

Seedling survival ranged from 88% in soil mixture B to 100% in mixtures D and F, and survival was 96.7% in unheated treatments compared to 88.1% in heated treatments (Figure 5). The interaction of soil mixture type and soil warming was not significant.

Seedling height: Average seedling height was 71.4 ± 23.5 mm. Seedling age and the interaction of soil mixture type × soil warming via heating mat had significant effects on *Ebenopsis* height, whereas soil type, soil warming, and the interaction of age × warming had marginal effects (Table 5).

**Figure 6:**
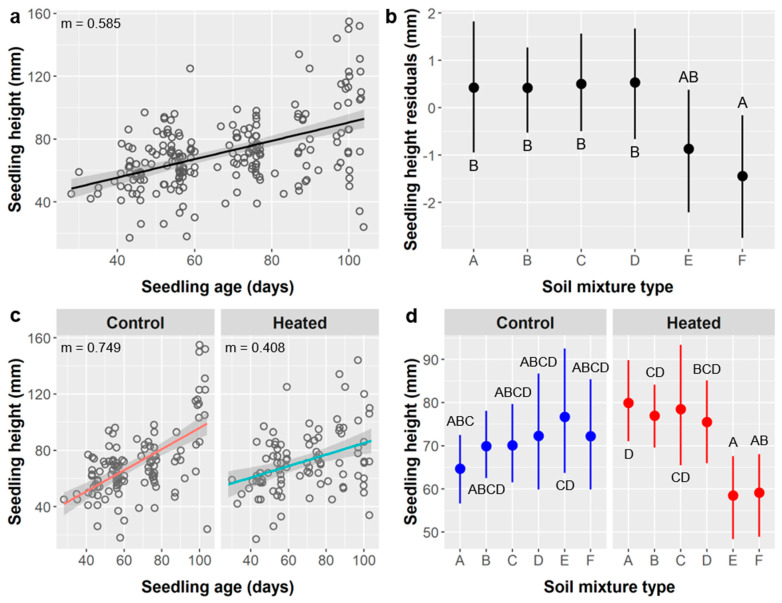
(**a**) Regression of *Ebenopsis ebano* seedling height by seedling age with the best-fit line (m = 0.585, R^2^ = 0.241). (**b**) Treatment means with 95% confidence intervals showing the effects of soil mixture type on *Ebenopsis* seedling height residuals. (**c**) Regression of seedling height by age in the control (R^2^ = 0.347) and heated (R^2^ = 0.138) soil warming treatments. The difference in slope reflects the marginally significant age × warming interaction and suggests that seedlings gained height faster in the unheated control treatment. This is paralleled by the marginally significant main effect of soil warming on seedling height (Table 5). (**d**) Treatment means with 95% confidence intervals for seedling height broken down by soil type and soil warming treatments. Seedling responses to warming differed by soil type (soil type × warming interaction), the details of which are described in the results. Capital letters in panels (**b**,**d**) denote the results of least square means post-hoc tests; treatments that share a letter were not significantly different.

Seedling height increased with age at a rate of 0.585 mm per day (Figure 6a). Although the main effect of soil type was marginally significant, post-hoc tests suggest that seedlings grown in soil types A (71.9 mm), B (72.9 mm), C (73.5 mm), and D (73.9 mm) were significantly taller than those in soil F (66.6 mm) (Figure 6b). Seedlings were marginally shorter in the soil warming treatment (70.7 mm) compared to the control (72.2 mm). Similarly, as signified by the marginal age × warming interaction, seedlings in the warming treatment gained height more slowly as they aged (0.408 mm/d) than those in the unheated control (0.749 mm/d) (Figure 6c). However, seedlings grown in different soil mixture types responded differently to the soil warming treatments (soil type × warming interaction). Specifically, seedlings grown in soil type D had similar heights in both the warmed (72.3 mm) and control treatments (75.5 mm), whereas seedlings were taller when heated for soil types B (76.9 vs. 69.9 mm) and C (78.5 vs. 70.1 mm) and significantly so for soil A (79.9 vs. 64.7 mm), but seedlings were shorter when heated in soil type F (59.2 vs. 72.2 mm) and significantly so in soil E (58.5 vs. 76.6 mm) (Figure 6d).

Seedling leaf abundance: *Ebenopsis* seedlings had an average of 12.3 ± 5.6 leaves at final data collection. Seedling age, SA treatment, soil type, and the interactions of age × soil type and soil type × warming significantly influenced leaf abundance, but the main effect of warming was not significant (Table 6).

**Figure 7:**
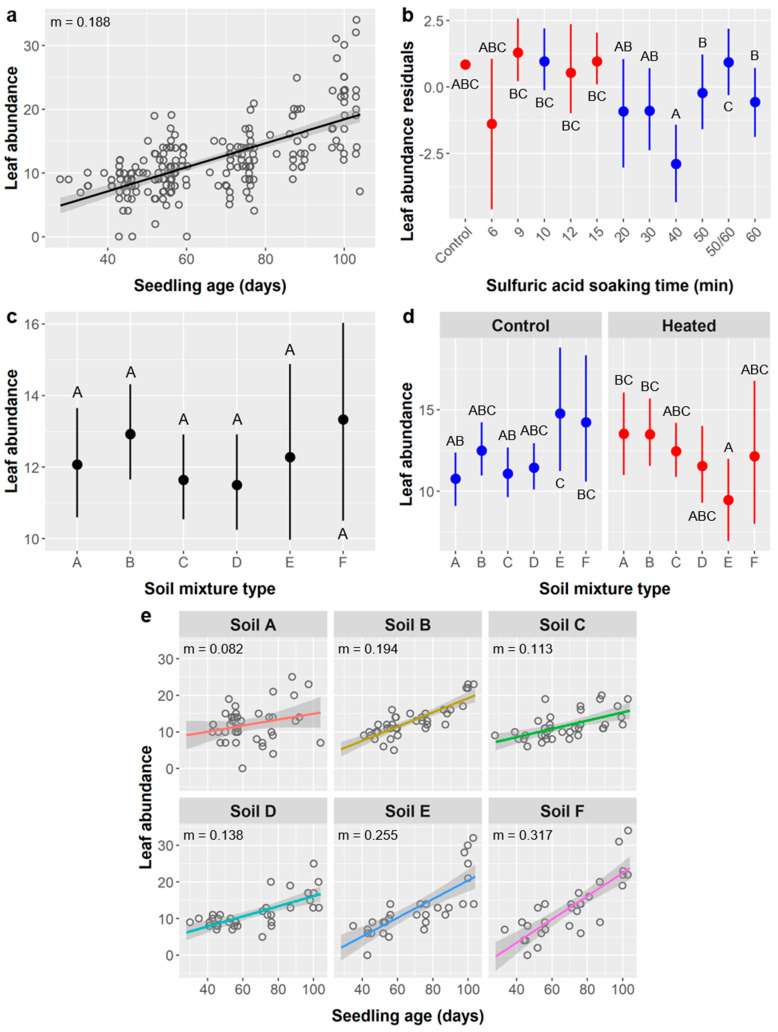
(**a**) Regression of *Ebenopsis ebano* leaf abundance by seedling age (R^2^ = 0.440). (**b**) Treatment means with 95% CIs showing the effects of sulfuric acid (SA) treatment on leaf abundance residuals. Points are colored by stirring treatment: red, stirred; blue, unstirred. The ‘50/60’ treatment refers to those seedlings that germinated first from the 50 and 60 min SA treatments and whose labels (tagged with a different label type and exposed to weathering the longest) became partially indecipherable. (**c**) Treatment means with 95% CIs showing the effects of soil type on leaf abundance. As seen here, differences detected by multifactor linear models like this ANCOVA are often not apparent when plotting results based on a single factor because they display variance that is explained by other factors. For this reason, we sometimes plot residuals instead, as in panel b, but they are less intuitive. (**d**) Treatment means with 95% CIs for leaf abundance broken down by soil type and warming treatments. Seedling responses to warming differed by soil type (soil type × warming interaction), the details of which are described in the results. (**e**) Regressions of leaf abundance by seedling age for each soil type. The age × soil type interaction shows that the relationship between leaf abundance and age differed among soil types and is reflected by the variance in slopes of the trendlines. Capital letters in panels **b**, **c**, and **d** denote the results of least square means post-hoc tests.

Leaf abundance increased with age by 0.188 leaves/d (Figure 7a) and was greater among seedlings whose seeds were exposed to shorter SA soak times (Figure 7b). There was a dichotomy between the shorter-duration stirred SA treatments (13.2 leaves) and longer-duration unstirred SA treatments (10.1 leaves), although the oldest seedlings in the 50 and 60 min unstirred treatments had leaf counts comparable to the stirred treatments (Figure 7b).

As seen above, the first *Ebenopsis* seedlings to germinate came from the 50 and 60 min SA treatments, and these individuals were tagged with a different label type that was exposed to weathering the longest and became partially indecipherable. When we could not distinguish between 50 and 60 min SA treatments at final data collection, we referred to these individuals as ‘50/60’ and considered them statistically as a separate group. Soil types D and C had the lowest leaf abundances (11.5 and 11.6 leaves, respectively) and types B and F the highest (12.9 and 13.3 leaves, respectively), but our post-hoc tests did not detect any significant differences between soil treatments (Figure 7c).

The significant interaction between soil type and soil warming reflects the fact that seedlings in soil types B, C, and D exhibited similar leaf abundance in both warming treatments, but soil warming produced leaf abundances that were higher in soil A (13.5 vs. 10.8 leaves), reduced in soil F (12.2 vs. 14.2 leaves), and significantly reduced in soil E (9.5 vs. 14.8 leaves) compared to the unheated control (Figure 7d). The relationship between seedling age and height also varied among soil treatments, as denoted by the significant age × soil type interaction and is illustrated by the difference in regression slopes when soil types are considered individually (Figure 7d). Slopes ranged from 0.082 and 0.113 leaves/d in soils A and C, respectively, to 0.255 and 0.317 leaves/d in soils E and F, respectively. Post-hoc tests showed a significant difference between the slopes of soils A and E.

### 2.5. Ebenopsis Seedling Belowground Growth

Root length: We analyzed roots of the oldest seedlings from the 50 and 60 min SA treatments, but ages still ranged from 83 to 106 days (mean 98.0 days), so we included age as a covariate in all root analyses. Root length averaged 350 ± 106 mm and was significantly affected by seedling age, SA treatment, soil type, soil warming, and the interactions of age × soil type and age × warming (Table 7).

**Figure 8:**
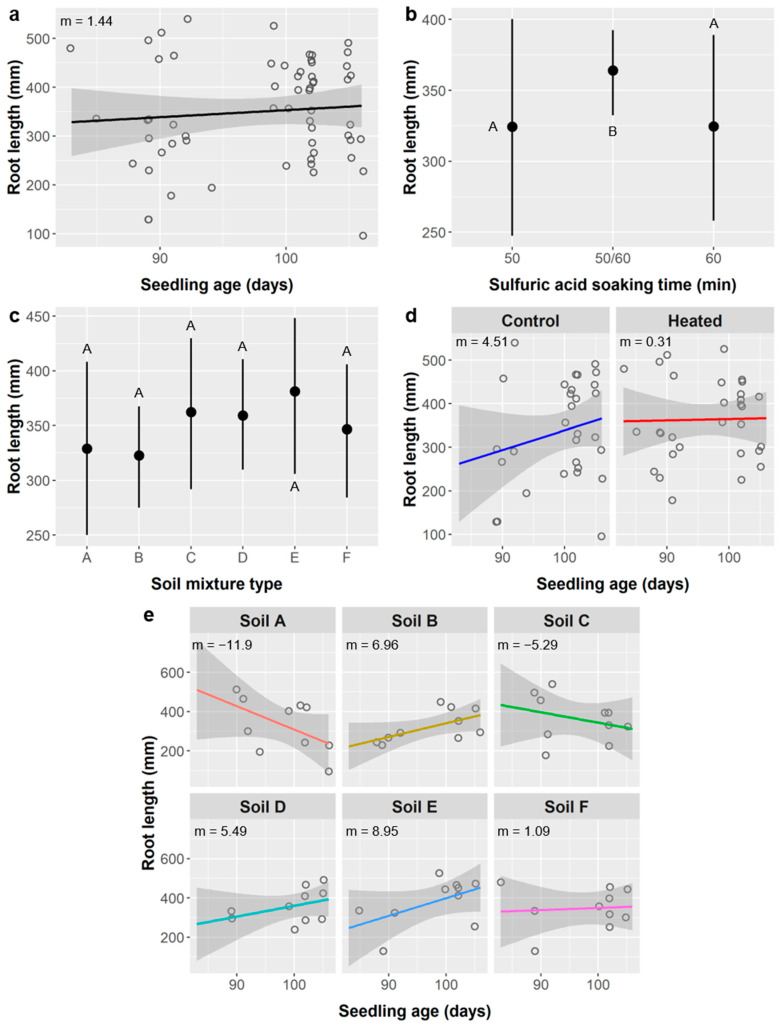
(**a**) Regression of *Ebenopsis ebano* root length by seedling age (R^2^ = 0.008). (**b**) Treatment means with 95% CIs showing the effects of sulfuric acid (SA) treatment on root length. (**c**) Treatment means with 95% CIs showing the effects of soil type on root length. (**d**) Regressions of root length by seedling age for each soil warming treatment. The age × warming interaction shows that soil warming influenced the relationship between root length and age and is reflected by the variance in slopes. (**e**) Regressions of root length by seedling age for each soil type. The age × soil type interaction shows that the relationship between root length and age differed among soil types and is reflected by the variance in slopes of the trendlines. Capital letters in panels b and c denote the results of least square means post-hoc tests.

The relationship between age and root length was weak (m = 1.44 mm/d, Pearson correlation = 0.089), but our Type III ANCOVA considered residual variation not explained by other model terms, and age had a significant effect on root length (*p* = 0.0210) (Figure 8a). Root length was significantly higher in the 50/60 SA treatment group (364 mm) than in the 50- or 60-min SA treatments (324 and 325 mm, respectively) (Figure 8b).

Seedlings grown in soil type E had the greatest root length (381 mm) and soil B had the lowest (323 mm), but, even though the main effect of soil type was significant (*p* = 0.0108), none of the soil type treatments were significantly different from any others in our post-hoc tests (Figure 8c). However, post-hoc tests did show that the mean root length in the soil warming treatment (363 mm) was significantly higher than in the unheated control (337 mm) (not shown). The relationship between age and root length also depended on the soil warming treatment (age × warming interaction), with a relatively strong positive relationship in the unheated control (m = 4.51 mm/d, Pearson r = 0.223) and essentially no relationship in the heated treatment (m = 0.31 mm/d, Pearson r = 0.023) (Figure 8d). Soil type also influenced the relationship between age and root length (Figure 8e), with the relationship ranging from negative in soil types A (m = −11.9 mm/d) and C (m = −5.29 mm/d) to weakly positive in soil F (m = 1.09 mm/d) and to more strongly positive in soils B (6.96 mm/d) and E (8.95 mm/d).

Root surface area: Root surface area averaged 120 ± 19.0 cm^2^ and was significantly influenced by seedling age, SA treatment, soil warming, and the interaction of age × warming, whereas soil type and the interaction of age × soil type had marginal effects on root surface area (Table 8).

**Figure 9:**
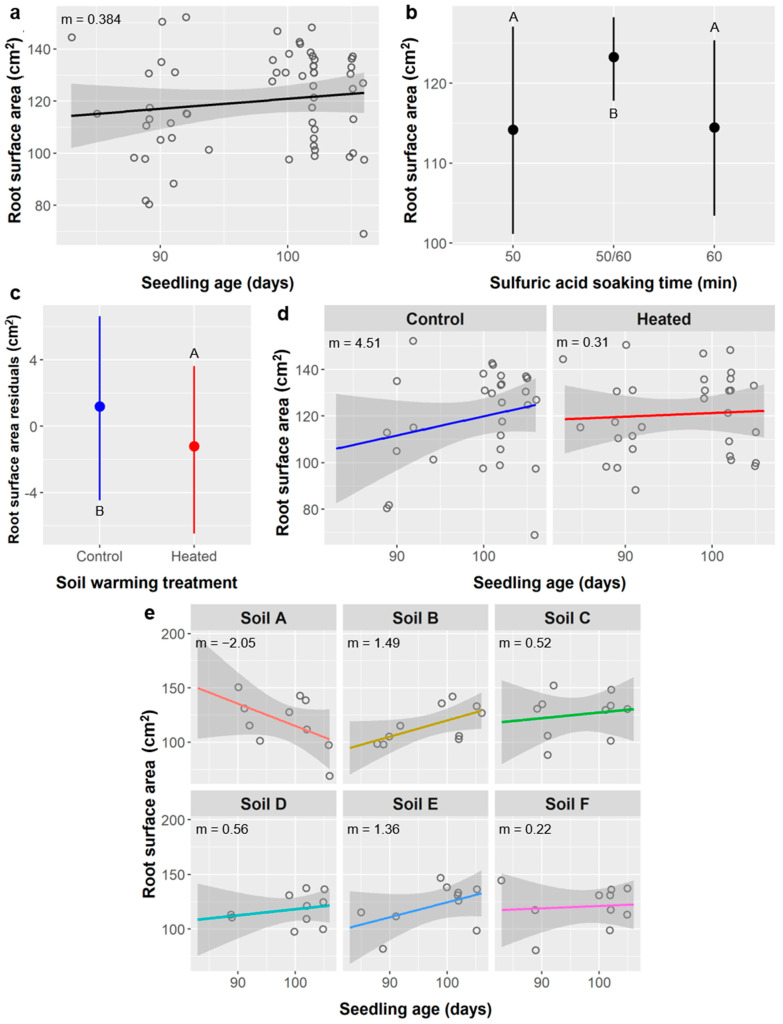
(**a**) Regression of *Ebenopsis ebano* root surface area by seedling age (R^2^ = 0.017). (**b**) Treatment means with 95% CIs showing the effects of sulfuric acid (SA) treatment on root surface area. (**c**) Treatment means with 95% CIs showing the effects of soil warming on root surface area residuals. (**d**) Regressions of root surface area by seedling age for each soil warming treatment. The age × warming interaction shows that soil warming influenced the relationship between root surface area and age and is reflected by the variance in slopes. (**e**) Regressions of root surface area by seedling age for each soil type. The age × soil type interaction shows that the relationship between root surface area and age differed among soil types and is reflected by the variance in slopes of the trendlines. Capital letters in panels b and c denote the results of least square means post-hoc tests.

Root surface area increased by 0.384 cm^2^ per day of seedling age (Pearson r = 0.132, *p* = 0.0132) (Figure 9a). Seedlings from the 50/60 SA treatment group had significantly greater root surface area (123 cm^2^) than seedlings grown from seeds treated with SA for 50 min (114 cm^2^) or 60 min (114 cm^2^) (Figure 9b). Soil type had a marginal effect on root surface area, which ranged from 116 cm^2^ in soil type B to 126 cm^2^ in soil C (Figure 9c). Root surface area was similar in the soil warming treatment (121 cm^2^) and the unheated control (120 cm^2^), but, after accounting for variance explained by other model terms, root surface area was significantly lower when heated by a difference of 2.4 cm^2^ (*p* = 0.0316) (Figure 9c). The relationship between root surface area and seedling age was significantly more strongly positive without soil warming (m = 0.815 cm^2^/d) than with warming (m = 0.157 cm^2^/d) (Figure 9d), and the relationship also varied among soil types and ranged from −2.05 cm^2^/d in soil type A to 1.49 cm^2^/d in soil B (Figure 9e), but the age × soil type interaction was marginal (*p* = 0.0833).

Root diameter: The diameter of sampled roots averaged 1.17 ± 0.28 mm. SA treatment, soil type, soil warming, and the interactions of age × soil type and age × warming significantly influenced root diameter, whereas seedling age and the soil type × warming interaction had marginal effects (Table 9).

**Figure 10:**
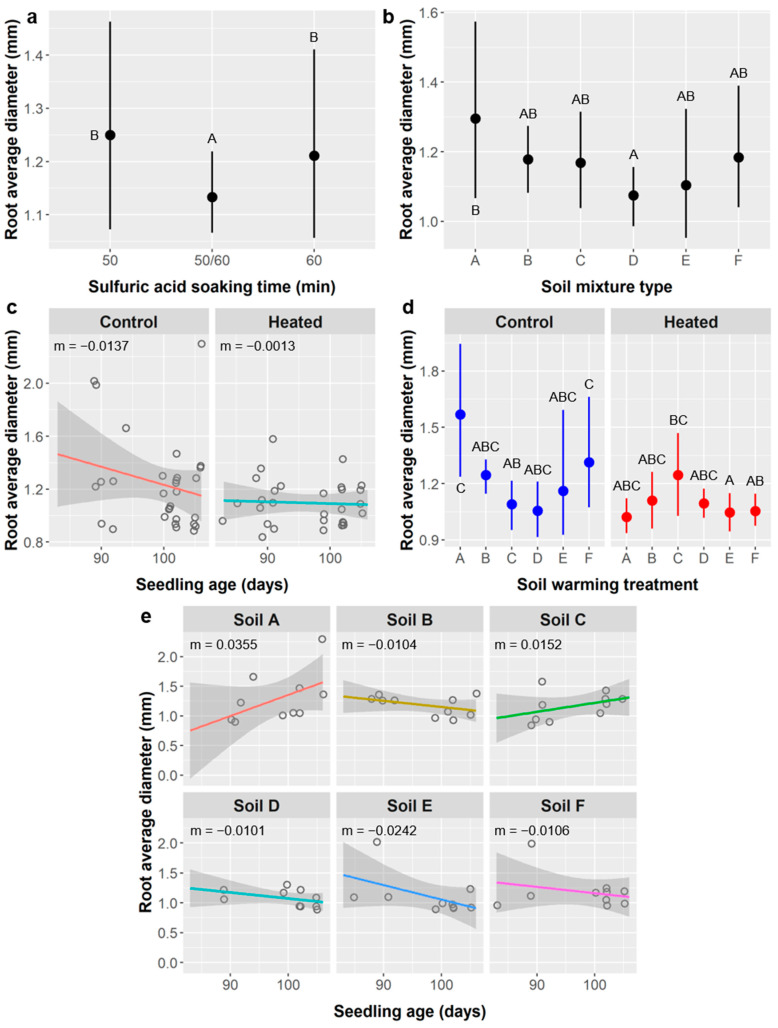
(**a**) Treatment means with 95% CIs showing the effects of sulfuric acid (SA) treatment on average root diameter. (**b**) Treatment means with 95% CIs showing the effects of soil type on root diameter. (**c**) Regressions of root diameter by seedling age for each soil warming treatment. The age × warming interaction shows that soil warming influenced the relationship between root diameter and age and is reflected by the variance in slopes. (**d**) Treatment means with 95% CIs for root diameter broken down by soil type and warming treatments. Seedling responses to warming differed by soil type (soil type × warming interaction), the details of which are described in the results. (**e**) Regressions of root diameter by seedling age for each soil type. The age × soil type interaction shows that the relationship between root diameter and age differed among soil types and is reflected by the variance in slopes of the trendlines. Capital letters in panels a, b and d denote the results of least square means post-hoc tests.

The mixed 50/60 group of seedlings had a significantly lower average root diameter (1.13 mm) than other seedlings from the 50 min (1.25 mm) or 60 min (1.21 mm) SA treatments (Figure 10a). Root diameter was significantly higher among seedlings grown in soil type A (1.30 mm) than in soil D (1.07 mm), while average root diameters from other soil types were intermediate (1.10−1.18 mm) (Figure 10b). Average root diameter was greater in unheated controls (1.24 mm) than in the soil warming treatment (1.10 mm). The relationship between seedling age and root diameter differed between soil warming treatments, with a negative relationship in the control (m = −0.0137 mm/d) and essentially no relationship when heated (m = −0.0013 mm/d) (Figure 10c). Average root diameter was similar in both soil warming treatments for soil types B and D, but was higher in heated soils for soil C (1.25 vs. 1.09 mm), lower when heated in soil E (1.05 vs. 1.16 mm), and significantly lower when heated in soils A (1.02 vs. 1.57 mm) and F (1.05 vs. 1.31 mm) according to post-hoc tests (Figure 10d), however, the soil type × warming interaction was only marginal in the ANCOVA. The relationship between seedling age and root diameter varied by soil type, with weak positive relationships for soils A (m = 0.0355) and C (m = 0.0152) and weak negative relationships for all other soil types (Figure 10e). 

Root volume: Average *Ebenopsis* root volume was 3.40 ± 0.43 cm^3^. Soil mixture type, soil warming, and the interaction of seedling age × soil type had significant effects on root volume (Table 10).

**Figure 11:**
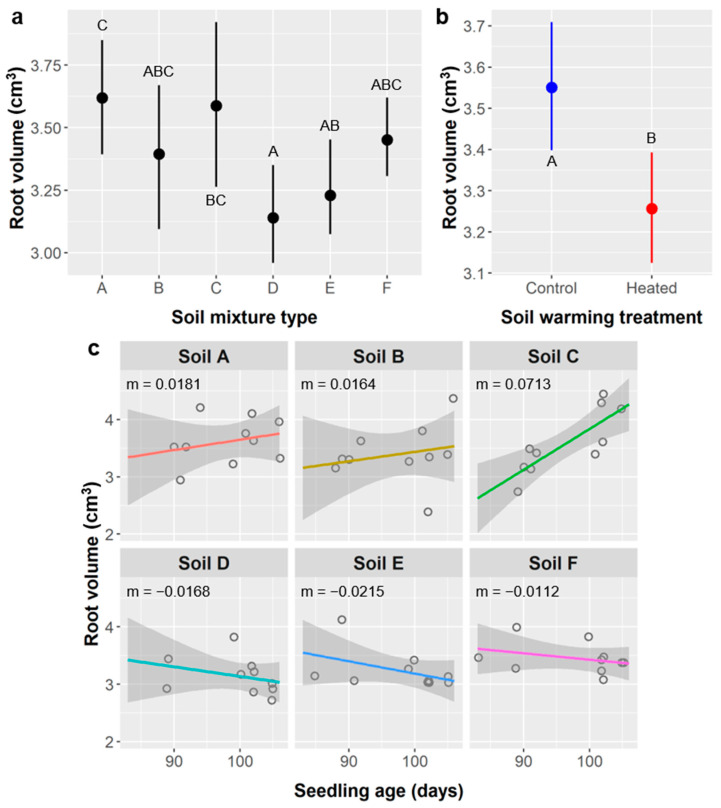
(**a**) Treatment means with 95% CIs showing the effects of soil type on root volume. (**b**) Treatment means with 95% CIs showing the effects of soil warming on root volume. (**c**) Regressions of root volume by seedling age for each soil type. The age × soil type interaction shows that the relationship between root volume and age differed among soil types and is reflected by the variance in slopes of the trendlines. Capital letters in panels a and b denote the results of least square means post-hoc tests.

Seedlings grown in soil types A and C had mean root volumes of 3.62 and 3.59 cm^3^, respectively, which were significantly higher than soil type D (3.14 cm^3^), while soils B, E, and F had intermediate root volumes (Figure 11a). Unheated controls had significantly greater root volume (3.55 cm^3^) than the soil warming treatment (3.26 cm^3^) (Figure 11b). The relationship between root volume and seedling age varied among soil types, ranging from strongly positive in soil C (m = 0.0713 cm^3^/d), to more weakly positive in soils A (0.0181 cm^3^/d) and B (0.0164 cm^3^/d), and to weakly negative in soils D, E, and F (−0.0168, −0.0215, and −0.0112 cm^3^/d, respectively) (Figure 11c).

Root tips: *Ebenopsis* seedlings developed an average of 1056 ± 496 root tips. Root tip abundance was significantly impacted by soil mixture type, soil warming, and the interactions of age × soil type and soil type × warming (Table 11).

**Figure 12:**
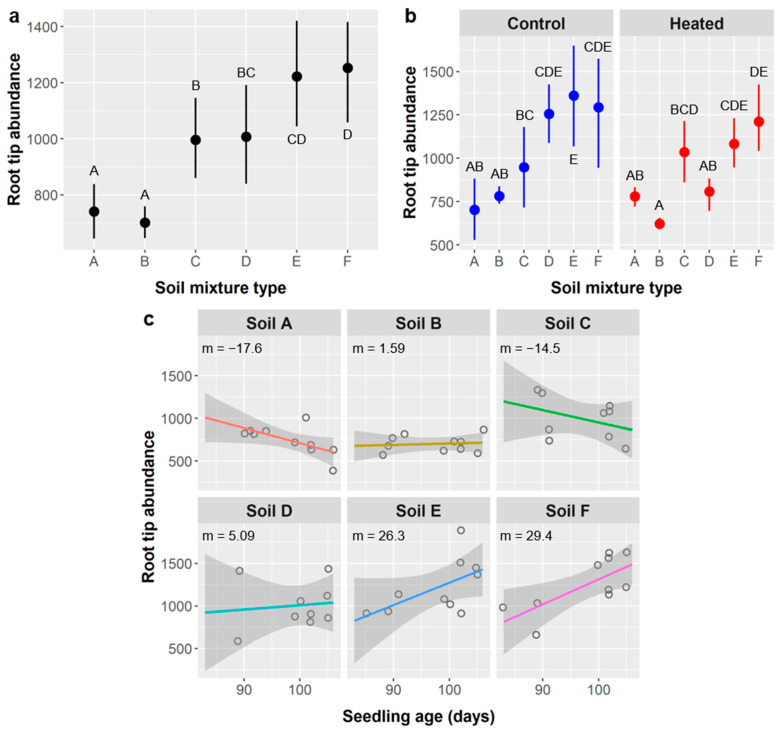
(**a**) Treatment means with 95% CIs showing the effects of soil type on root tip abundance. (**b**) Treatment means with 95% CIs for root tips broken down by soil type and warming treatments. Seedling responses to warming differed by soil type (soil type × warming interaction), the details of which are described in the results. (**c**) Regressions of root tips by seedling age for each soil type. The age × soil type interaction shows that the relationship between root tips and age differed among soil types and is reflected by the variance in slopes of the trendlines. Capital letters in panels a and b denote the results of least square means post-hoc tests.

Seedlings grown in soil types A and B had significantly fewer root tips than seedlings in any other soil types (741 and 702 tips, respectively), whereas seedlings from soils E and F had significantly more root tips than any other soil types (1222 and 1252 tips, respectively), with the exception that soils D and E were not distinguishable (Figure 12a). Soil warming significantly reduced root tip abundance compared to the unheated control (923 vs. 1188 tips), but its effect depended on soil type. Root tip abundance was similar in both warming treatments for soil types A, C, and F, but was lower in heated treatments for soils B (621 vs. 782 tips) and E (1083 vs. 1360), and significantly so for soil D compared to the control (808 vs. 1571) (Figure 12b). Soil type also influenced the relationship between seedling age and root tip abundance, with soil types E and F exhibiting positive relationships (m = 26.3 and 29.4 tips/d, respectively), soils B and D neutral relationships (1.59 and 5.09 tips/d, respectively), and soils A and C negative relationships (−17.6 and −14.5 tips/d, respectively) (Figure 12c).

**Figure 13:**
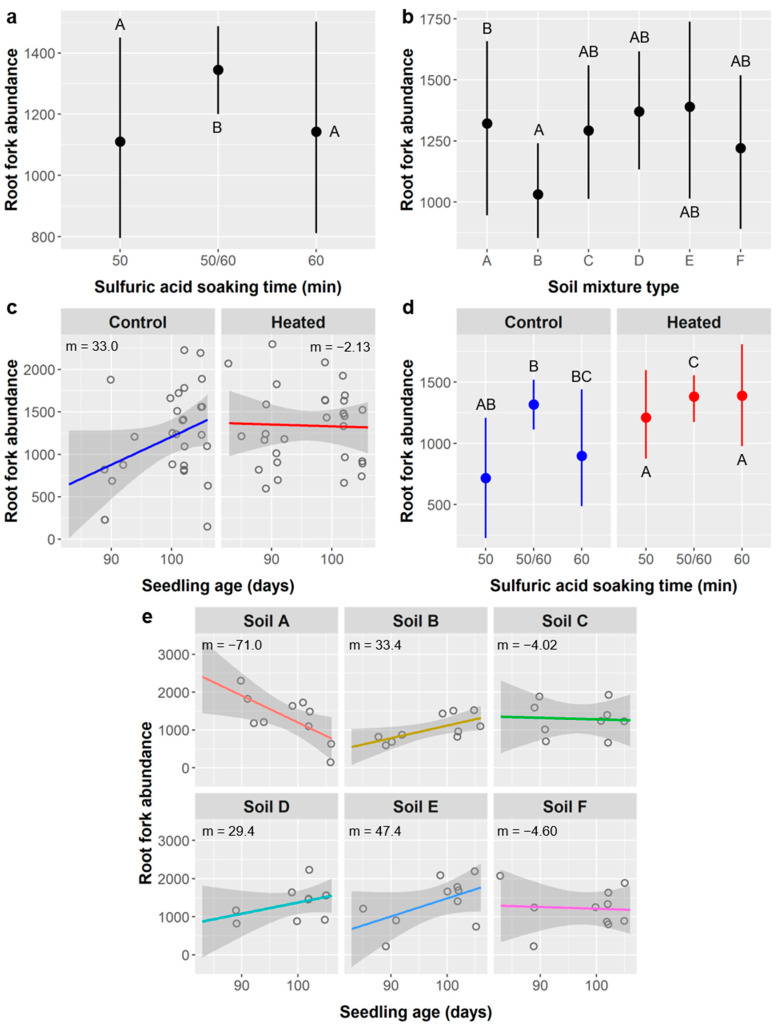
(**a**) Treatment means with 95% CIs showing the effects of sulfuric acid (SA) treatment on root fork abundance. (**b**) Treatment means with 95% CIs showing the effects of soil type on root forks. (**c**) Regressions of root fork abundance by seedling age for each soil warming treatment. The age × warming interaction shows that soil warming influenced the relationship between root forks and age and is reflected by the variance in slopes. (**d**) Treatment means with 95% CIs for root fork abundance broken down by SA and warming treatments. Seedling responses to warming differed by SA treatment (SA × warming interaction), the details of which are described in the results. (**e**) Regressions of root forks by seedling age for each soil type. The age × soil type interaction shows that the relationship between root forks and age differed among soil types and is reflected by the variance in slopes of the trendlines. Capital letters in panels a, b and d denote the results of least square means post-hoc tests.

Root forks: *Ebenopsis* roots had an average of 1271 ± 508 forks across all treatments. Seedling age, SA treatment, soil mixture type, soil warming, and the interactions of age × soil type, age × warming, and SA treatment × warming all significantly impacted root fork abundance (Table 12). Fork abundance increased by 8.46 forks per day of seedling age (Pearson r = 0.109, *p* = 0.0060) and was significantly higher in the 50/60 SA treatment group (1345 forks) than in the 50 or 60 min SA treatments (1111 and 1143 forks, respectively) (Figure 13a). Seedlings from soil type A had significantly more root forks (1322) than those from soil B (1032 forks), while fork abundances in other soil types were comparable to type A (1220–1391 forks) but were highly variable (Figure 13b).

Soil warming significantly increased fork abundance from 1203 in the control to 1336 forks in the heated treatment, and influenced the relationship between root forks and seedling age, which was positive in the control (m = 33.0 forks/d) and neutral in the heated treatment (m = −2.13) (Figure 13c). In the unheated control, the three SA treatments had indistinguishable means, but, in the heated treatment, the 50/60 group produced significantly more root forks than the 50- or 60-min SA treatments (SA × warming interaction) (Figure 13d). The relationship between root fork abundance and seedling age varied significantly among soil types and was negative in soil type A (−71.0 forks/d), neutral in soils C and F (−4.02 and −4.60 forks/d, respectively), and positive in soils B, D, and E (33.4, 29.4, and 47.4 forks/d, respectively) (Figure 13e).

Root crossings: In the root analyses performed using the WinRHIZO scanner and software, root crossings refer to instances where separate roots overlap in the 2D projection of the 3D root system. We observed an average of 169 ± 95 root crossings in *Ebenopsis* seedlings. Root crossing abundance was significantly influenced by seedling age, SA treatment, soil warming, and the interactions of age × warming and SA × warming, and was marginally influenced by soil type and the interaction of age × soil type (Table 13).

**Figure 14:**
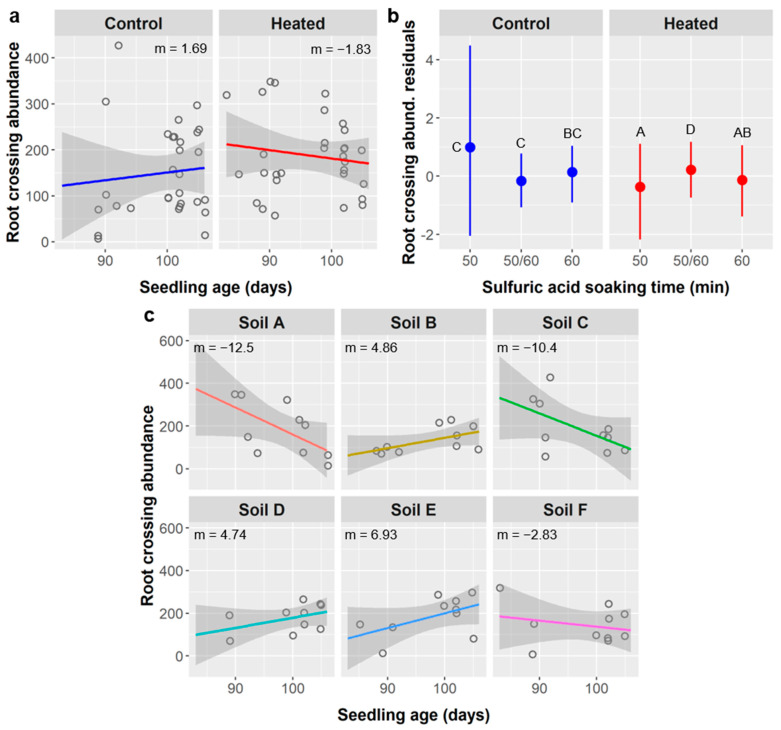
(**a**) Regressions of root crossing abundance by seedling age for each soil warming treatment. The age × warming interaction shows that soil warming influenced the relationship between root crossings and age and is reflected by the variance in slopes. (**b**) Treatment means with 95% CIs for root crossing abundance residuals broken down by SA and warming treatments. Seedling responses to warming differed by SA treatment (SA × warming interaction), the details of which are described in the results. (**c**) Regressions of root crossings by seedling age for each soil type. The age × soil type interaction shows that the relationship between root crossings and age differed among soil types and is reflected by the variance in slopes of the trendlines. Capital letters in panel b denote the results of least square means post-hoc tests.

Root crossings had a weak but significant negative relationship with seedling age (m = −1.05 crossings/d, Pearson r = −0.072, *p* = 0.0053). Seedlings grown from seeds in the 50/60 SA treatment group had 169 root crossings, which was intermediate between the 50- and 60 min SA treatments with 176 and 161 crossings, respectively. However, post-hoc tests that accounted for variance explained by other model terms found that root crossing residuals were significantly higher in the 50/60 treatment than in the 50 min SA treatment. Root crossings were significantly more abundant in the soil warming treatment (188 crossings) than in the unheated control (150 crossings). The relationship between crossings and seedling age was positive in the unheated control (m = 1.69) but negative in the warmed treatment (m = −1.83) (Figure 14a). Residual root crossing abundances were similar across all SA treatments in the unheated control, but the 50/60 SA group had significantly higher root crossing residuals than the 50- or 60-min SA treatments in the warming treatment (Figure 14b). Soil type had only a marginal effect on the relationship between root crossing abundance and seedling age, but the variability among soil types was notable, ranging from strongly negative in soil types A and C (m = −12.5 and −10.4 crossings/d, respectively), to weakly negative in soil F (−2.83 crossings/d), and to positive in soils B, D, and E (4.86, 4.74, and 6.93 crossings/d, respectively) (Figure 14c).

## 3. Discussion

Seed and seedling availability are the most limiting factors for restoration of high-value Tamaulipan thornforest habitat in the Lower Rio Grande Valley of south Texas. Seed supply depends on wild collection, which is labor-intensive and requires significant expertise. This challenge is compounded by low or highly variable germination rates of many thornforest species, and by knowledge gaps regarding the germination requirements and best horticultural practices for propagating thornforest species from seed. This study addressed key knowledge gaps and focused on three ecologically important thornforest species (*Ebenopsis*, *Cordia*, and *Zanthoxylum*) but encountered challenges common in thornforest seedling production. Germination rates were so low for two focal species that experiments yielded limited or no useful results. However, all *Ebenopsis* experiments were successful, and our investigation of gibberellic acid treatments and heat cracking of seed coats for *Cordia* yielded valuable data and compelling results.

### 3.1. Ebenopsis ebano (Texas ebony)

*Ebenopsis* seeds responded very strongly to sulfuric acid (SA) treatments. This was consistent with prior studies [33] and current recommendations [22,31], and reflects germination requirements of other Fabaceae species with thick seed coats like *Ebenopsis* [32]. However, we identified an optimum SA soak time of 40–60 min, which is longer than the currently recommended SA soak time of 30–35 min [31] and can be easily replicated by commercial growers (Figure 1). Stirring *Ebenopsis* seeds during SA treatment can accelerate the scarification process and may reduce molding, but it is not necessary, and it increases the time required for germination (Figure 2).

Gibberellic acid (GA) alone did not improve *Ebenopsis* germination regardless of the dose tested (from 5–500 mg/L), but, mechanistically, this may be because the thick seed coat of *Ebenopsis* prevented GA from reaching the embryo (Figure 3). Thus, we cannot rule out the possibility that GA treatment could promote *Ebenopsis* germination in combination with other seed treatments that increase permeability of the seed coat, such as SA treatment. The need to overcome consecutive dormancy mechanisms in this fashion is well established in the literature [27,32]. However, observed germination rates of over 90% in the most favorable SA treatments suggest the effects of GA are likely to be relatively weak compared to those of SA, but *Ebenopsis* germination is often lower even with SA treatment [22,31], and GA treatment could have a larger effect in these cases. The effects of combining SA and GA treatments merit further investigation. Since the optimal SA soak time depends on seed coat thickness, which varies spatiotemporally, future study is also merited into whether 40–60 min is optimal across years and populations.

Potential impacts of seed treatments on seedling performance are important to consider, especially if negative effects could negate or exceed a treatment’s positive effects on germination. Importantly, SA treatments had minimal impacts on post-germination performance. SA treatment did not affect seedling survival or height (Table 4 and Table 5), and it had a weak effect on leaf abundance (Table 6, Figure 7b). Several root metrics did depend on SA treatment, including root length (Figure 8b), surface area (Figure 9b), diameter (Figure 10a), fork abundance (Figure 13d), and crossing abundance (Figure 14b), all of these except root diameter were higher in the 50/60 SA treatment group. For leaves, after considering the effects of other factors, the shorter-duration stirred SA treatments had about two more leaves than the longer-duration unstirred SA treatments, which is a small but notable difference. Nevertheless, survival was not affected by SA treatment, and these differences are not significant enough to justify avoiding longer SA treatments if they confer ca. 20% higher germination of *Ebenopsis* seeds.

Furthermore, in these cases of leaf abundance and root metrics, the link between SA treatment and seedling performance may actually be driven by the relationship between seedling age and performance. For leaves, the stirred SA treatments and shorter unstirred SA treatments germinated significantly later and thus had younger seedlings at the time of data collection (mean age = 52 days for stirred SA treatments, 42 d for 10 min unstirred, and 74 d for ≥20 min unstirred). For all root metrics, the 50/60 SA group were seedlings from the 50 or 60 min unstirred SA treatments that were planted earlier and whose labels degraded, and were thus ca. 12.5 days older (mean age = 90 d for 50 min SA treatment, 89 d for 60 min SA, and 102 d for the 50/60 SA group).

**Figure 15:**
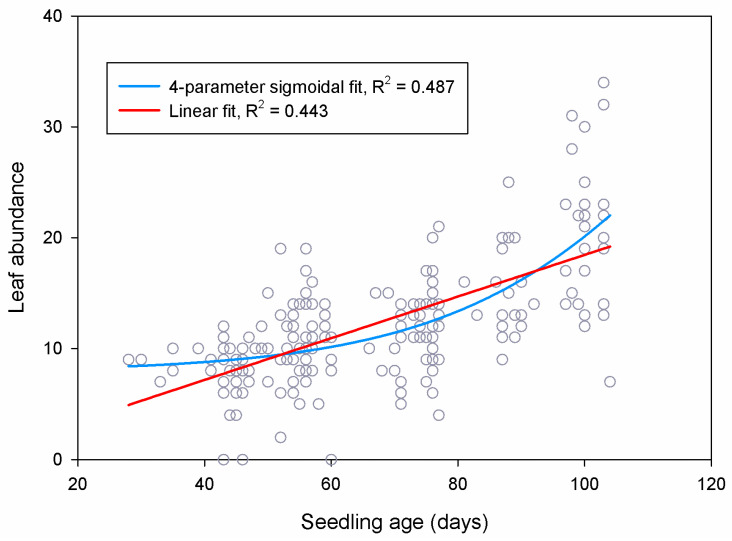
Linear (red) and nonlinear regression using a 4-parameter sigmoidal function (blue) of *Ebenopsis ebano* leaf abundance by seedling age. Both models were significant (*p* < 0.0001 for both), but the nonlinear curve fit the data slightly better as demonstrated by the differences in their coefficient of determination (R^2^) values.

In theory, differences in age among treatment groups should be accounted for statistically by including seedling age in our models, which we did. However, this may fail to explain all the variance driven by seedling age if the relationship between age and performance is more complex than represented by our linear models, i.e., if the relationship is nonlinear. This appears to be the case in our data, and this is supported by prior studies documenting ontogenetic variation in plant growth strategies, with different patterns of carbon allocation to roots, stems, and leaves at different life stages [57,58,59]. We fit a 4-parameter sigmoidal function to the relationship between leaf count and seedling age and performed a nonlinear least-squares regression using SigmaPlot 11 (Systat Software, Inc., Chicago, IL, USA). The sigmoidal relationship was significant (*p* < 0.0001) and explained more variability (R^2^ = 0.487) than the linear model (R^2^ = 0.443) (Figure 15).

The sigmoidal curve was relatively flat for seedlings aged ca. 20–60 days, which suggests a growth pattern emphasizing early foliation, which is consistent both with our observations and the typical growth strategies of plants like *Ebenopsis* that have large seeds with nutritional stores capable of fueling rapid growth immediately post-germination. Accelerated foliation after 60 days, as shown, is also consistent with ontogenetic growth strategies because plants become capable of faster absolute growth as they grow and their total photosynthetic capacity increases.

The relationships between SA treatment and root length, surface area, diameter, forks, and crossings are less easily explained as an effect of age because the linear relationships between these root metrics and seedling age are weak (Figure 8, Figure 9, Figure 10, Figure 13 and Figure 14). There are three reasons why these relationships are weaker than those between root metrics and the differently-aged SA treatment groups: (1) a much shorter range of ages are represented (83–106 days); (2) there was high variability in root metrics that overshadowed age-related trends; (3) there were significant interactions between age and other factors like soil type and soil warming. Thus, the effects of SA on root metrics are probably more an artefact of our experimental design than they are evidence that SA seed treatments altered *Ebenopsis* seedling performance.

Additionally, weakening the relationship between root morphology and age is the observation that, for root length, surface area, forks, and crossings, there was less variance at higher ages (and thus among the 50/60 SA group, specifically), and the peak values for older seedlings were comparable to the peak values among younger seedlings (e.g., Figure 8a,b and Figure 9a,b). Taken together, this is evidence that the older seedlings became space-limited (root-bound) and their root growth and development were likely altered or restricted in some way, which is not surprising for 3-month-old seedlings being grown in ca. 200 mL of soil. Even if age is not the underlying factor driving differences between SA treatments, the effects of SA on root metrics were weak compared to the effects of soil type and soil warming. Thus, for both leaf abundance and root morphology, we found no compelling reasons to limit the use of SA to promote germination of *Ebenopsis* seedlings.

Soil type and soil warming were the only factors to influence survival, and they had the strongest effects on above- and belowground growth. Soil types D (50% peat, 25% sand, 25% vermiculite) and F (50% peat, 20% topsoil, 20% vermiculite, 10% perlite) were the only mixtures to have 100% survival in both soil warming treatments (Figure 5). This may be because they had the greatest water-holding capacity, but soil F also had the lowest bulk density. Local topsoil is a sandy clay loam that drains quickly but holds more water than sand or perlite [60], which are used horticulturally to improve drainage and soil aeration. Peat moss and vermiculite hold the most water and are often used for that reason. Soils D and F had combined totals of 75% and 70%, respectively, of peat plus vermiculite, with the next highest mixtures containing 50%, as found in soil types A (50% topsoil, 50% vermiculite), C (50% peat, 25% sand, 25% topsoil), and E (50% peat, 25% perlite, 25% sand). The mixtures with 50% peat + vermiculite had intermediate survival rates, whereas soil type B (50% topsoil, 25% perlite, 25% vermiculite) likely had the lowest water-holding capacity and exhibited the lowest seedling survival (Figure 5).

Bulk density is another important soil property that influences root growth and varied considerably among our soil mixture treatments. Soil types A, B, D, and E all had bulk densities of approximately 0.690 g/cm^3^ (range: 0.675–0.702 g/cm^3^; see Methods), whereas soil F was least dense (0.480 g/cm^3^), and soil C was most dense (0.892 g/cm^3^). In the hot and semi-arid region of south Texas, water availability and water stress are of central importance to plant survival and performance, so it is reasonable that survival was governed by edaphic factors that influenced water availability (e.g., water-holding capacity and evaporation rate) and root development (e.g., bulk density, porosity). It follows mechanistically that survival was lower with soil warming (Table 5, Figure 5) because heated treatments would have had a higher evaporation rate and possibly higher transpiration.

The relationships between seedling performance and soil type and warming were more nuanced. Generally, aboveground growth decreased and belowground growth increased when soils were warmed via heating mats, but soil type had idiosyncratic effects on performance and often influenced seedling responses to soil warming. Greater belowground growth at the cost of lower aboveground growth, as observed, is a common response to water stress and is consistent with observed survival patterns. Mechanistically, as water stress increases, most plants will preferentially allocate more carbon to root growth (produce more or larger roots) to increase their capacity to uptake water, and a higher root:shoot ratio can increase tolerance of water stress by reducing transpirational losses in conjunction with greater water absorption capacity [57]. Many of the specific responses to soil warming described above reflect this general pattern.

Other responses to warming reflect the same stress response. In unheated controls, we observed positive relationships between seedling age and root length, surface area, fork abundance, and crossing abundance, but in the soil warming treatments we saw neutral or weakly negative relationships between seedling age and the same variables (Figure 8d, Figure 9d, Figure 13c and Figure 14a). One would expect a positive relationship in both treatments and higher values in the warming treatment if warming increased water stress and plants responded by producing more roots, but not if belowground growth was limited by container size and seedlings had become space-limited, as we demonstrated above was likely the case. Importantly, the mean values for these root metrics in warming treatments are at the upper limit of the ranges of values observed in the unheated controls, which suggests seedlings in the control approached the container-imposed upper limit on root size as they reached the upper limits of age, but seedlings in the warming treatment had already reached that container-imposed limit by the lowest ages in the range analyzed. This is further evidence that container size affected seedling growth and root development.

We saw a similar but opposite pattern for root average diameter (Figure 10c), but this is part of the same water stress response mechanism. Decreased root diameter can be consistent with both increased root growth and the water stress response because only secondary growth increases diameter, and it will not occur unless there is enough fine (low diameter) root mass to provide required water absorption. Most water is absorbed by fine roots with high surface area to volume ratios, so having relatively more fine roots (and thus a lower average root diameter) can improve water absorption capacity.

Soil type had more idiosyncratic effects on root metrics and often influenced relationships with age and seedling responses to soil warming. Relative differences in seedling performance attributable to the main effects of soil type are summarized in Table 14. If we consider marginal effects (0.1 > *p* ≥ 0.5), relative aboveground performance was highest in soil B, high in soil A, and mixed or intermediate in soils C, D, E, and F, whereas belowground performance was highest in soils A and C, intermediate in soils D, E, and F, and lowest in soil B. We expected the opposite pattern for seedling performance in soil B since it had the lowest water-holding capacity and seedlings typically allocate more carbon belowground in response to water scarcity. However, mechanistically, if soil B was least suitable for *Ebenopsis* root growth, we would expect a “top-heavy” growth pattern and reduced survival, which we observed (survival was lowest in soil B; Figure 5).

Soil mixtures with more peat moss (types C–F) had mixed results, which is somewhat surprising because high peat content usually promotes root growth horticulturally. Soil C had the highest bulk density, and high bulk density impedes root growth, but even the maximum bulk density of soil mixtures utilized in these experiments (0.892 g/cm^3^) was well below the range in which bulk density begins to inhibit root growth [61]. Soil F had the lowest bulk density, but its belowground performance was intermediate. These findings suggest that the general benefits of reduced bulk density (e.g., increased porosity, aeration, and water holding capacity) were present in all the soil mixture types utilized.

The overall leader in relative performance was soil A, which, notably, is the formulation recommended by USFWS. Soil A was half native topsoil and half vermiculite, which basically serves to improve the water-holding capacity of native soil without altering its chemistry (as peat does). This likely reflects adaptation by native plants to their native soils and may suggest the presence of important beneficial organisms in native soil. Whether the benefits of soil A translate to other Tamaulipan thornscrub species merits investigation.

Table 15 summarizes the nature of the linear relationships between seedling age and *Ebenopsis* performance variables for each soil type. The prevalence of negative relationships between age and belowground performance metrics for soil A, and to a lesser degree soil C, suggests root growth became spatially limited most rapidly in these soil types, and may suggest that there was root growth outside of the containers that was not harvestable. Alternatively, but by the same logic, the prevalence of positive relationships between age and belowground metrics for soils B and E suggests roots were slowest to become spatially limited in these soil types, which is consistent with the hypothesis that soil mix B was least conductive for *Ebenopsis* root growth.

Finally, Table 16 summarizes the effects of soil warming on *Ebenopsis* performance in each soil type. In soils E and F, warming had a negative effect on most performance metrics, and both contained 50% peat plus a relatively large proportion of drainage promoting materials (i.e., perlite, sand, or vermiculite). Heating likely had a relatively strong effect on increasing evaporation from these soil treatments. This would increase water stress but also promote root growth by triggering the water stress response, and these low density, high porosity soils should provide highly favorable conditions for root growth. The result may be belowground conditions that promote root growth while growth is limited by water availability, which could explain the mixed performance results (Table 14) and variable relationships with age (Table 15) observed for soils E and F. Soil warming had few effects in soils B, C, and D. 

Interestingly, warming had positive aboveground effects and a strong negative effect on root diameter in soil A. Decreased average root diameter likely reflects a greater abundance of fine root mass in this context, which is supported by Table 14 and the fact that aboveground performance increased. Mechanistically, higher evaporation with warming could have driven an increase in fine root mass by triggering the water stress response, and it could also reflect greater activity of beneficial soil organisms triggered by warming. Positive effects conferred by beneficial soil organisms might be observed most strongly in soil A because it was the most similar to native soil physically and chemically.

Further study is merited to differentiate between the roles of water stress and water availability in driving these patterns versus the effects of soil bulk density, soil porosity, and other physicochemical attributes like soil pH. The role of soil microorganisms in driving seedling survival and performance is also worth investigating, including whether natural or commercial soil inoculants can boost performance. 

These germination and rearing practices are not confined to *Ebenopsis*. There are numerous thornscrub species in the Fabaceae with seeds and growth habits comparable to *Ebenopsis*, including four *Acacia* species. Some of these other legumes have exhibited low germination rates and substantially slower growth than *Ebenopsis*. Thus, these insights into *Ebenopsis*’s seed treatment and rearing methods can provide a useful foundation for future investigations into the propagation of other thornforest species.

### 3.2. Cordia boissieri (Mexican olive)

For *Cordia*, we posit that treating seeds with 100 mg/L GA is optimal because increasing germination likelihood is most important, and this dose offers a large increase in germination from ca. 35% to over 60% (among cracked seeds) while imposing only a modest delay in germination timing. In practice, if germination takes 12 days instead of 9, as our data suggest for this dosage, this would impose only a minor inconvenience and is much less important than nearly doubling the likelihood of germination. Furthermore, the observed differences in germination timing were not statistically significant according to our ANOVA (Table 3), which further supports the notion that differences in germination likelihood are paramount for *Cordia*.

Like *Ebenopsis*, *Cordia* seeds have a hard exterior, but instead of being a thickened seed coat, it is the hardened endocarp or pit of the fruit, which is a drupe like a peach or cherry. Physical dormancy due to an impermeable seed coat has not been recognized in *Cordia*’s family, the Boraginaceae, nor is it one of the forms of dormancy employed by the most familiar drupes of the Rosaceae [25]. Nevertheless, our data suggests that physically breaking down the endocarp of *Cordia* may enhance the likelihood of germination (Figure 4). We cannot be sure, however, that the observed increase in germination was entirely due to the physical cracking of the endocarps achieved by desiccating the outer layer because this ‘heat cracking’ treatment was produced by accident on all the seeds from one population of *Cordia* trees. It is possible the differences in germination between cracked and not cracked treatments are due to differences in viability between the populations tested. Unfortunately, seeds were highly limited, so we could not heat crack more seeds from additional populations to eliminate this confounding factor. The effects of heat cracking *Cordia* seeds merits further study.

Furthermore, this uncertainty could have been partially, if not entirely, resolved by estimating the viability of all populations through tetrazolium testing or a similar approach. Tetrazolium testing in its own right is worthwhile in studies of thornscrub species because data on their seed viability is exceedingly limited, and this makes it currently impossible to link seed viability with other factors [22]. For these reasons, among others, we argue that seed viability of thornscrub species should always be tested (e.g., via tetrazolium), especially alongside experimental factors in future studies of germination.

Among uncracked seeds, GA concentrations over 100 mg/L further increased *Cordia* germination, but the GA × cracking interaction raises mechanistic uncertainties. In the uncracked treatment, the increase in germination is neatly proportional to GA concentration, but, in the cracked treatment, there is no such proportional relationship. If the viability of seeds was similar among cracked and not cracked populations, the germination patterns observed would suggest that germination was largely driven by imbibition of water rather than GA dosage. In this scenario, if a limited amount of water penetrated *Cordia* seeds in the uncracked treatment, the GA dose may matter in those cases and could have produced the results observed. Alternatively, if cracked seeds had higher viability, that alone could explain observed differences in germination between the two groups, but it would not explain why germination was proportional to GA concentration in the uncracked seeds but not the cracked seeds. Instead, this might suggest that *Cordia* populations differ in both base viability and in their responses to GA.

In practice, if observed patterns hold true, GA treatment should be a useful means to enhance *Cordia* germination, and the heat cracking technique we discovered could prove exceptionally useful and cost-effective at both small and large scales of production.

### 3.3. Zanthoxylum fagara (colima)

*Zanthoxylum* had exactly one germinant across all treatments. While this was obviously insufficient for analyses, we can deduce several things from this lack of germination. It is possible that our *Zanthoxylum* seed was viable, but that none of our treatments effectively broke its dormancy, or that some did so but also damaged the embryos in the process. Without testing seed viability, we cannot be sure, and this reaffirms our conclusion that future research into thornscrub species germination should include tetrazolium testing or some other approach to estimating viability.

It is much more likely that the *Zanthoxylum* seeds tested simply had exceedingly low viability. Low viability could have resulted for several reasons that are not mutually exclusive. First, we could have exceeded *Zanthoxylum*’s maximum seed storage time prior to planting. Second, immature seeds may have been collected. Third, the seeds could have been mature but not viable due to environmental stress during seed development, extensive seed predation, or some other ecological factor. Fourth, the seeds could have been viable when collected but rendered not viable at some point during their handling, processing, or storage; for example, if a critical temperature threshold was exceeded. Other explanations are possible as well.

Of these possible causes for near-zero *Zanthoxylum* seed viability, we believe the first is most likely. Recent findings suggest that fresh *Zanthoxylum* seed is required for propagation [22]. Although the exact duration of post-harvest viability is not clear, it is on the order of days to weeks and probably not more than several months. The storage time of our *Zanthoxylum* seed likely exceeded the viability window. We can largely rule out the second possible cause because *Zanthoxylum* fruits change color as they ripen and dehisce upon maturity, and seeds were collected from fruits that had dehisced. However, fruits were collected by harvesting branches with abundant fruits that were later processed. It is possible that the seeds retained on the branches at the time of collection were those that did not readily disengage from their dehisced fruits, which could be associated with malformation and/or nonviability of the seeds left behind. The exact nature of *Zanthoxylum*’s post-harvest seed viability window and storage techniques that might extend that window merit further investigation. Additionally, worth studying are the viability of *Zanthoxylum* seeds at different stages of fruit development and dehiscence, the viability of seeds from trees and populations exposed to different environmental conditions, and the prevalence and impacts of seed predation on *Zanthoxylum* seeds.

## 4. Materials and Methods

### 4.1. Focal Thornscrub Plant Species

*Ebenopsis ebano* (Texas ebony in English or ebano in Spanish; Fabaceae) is a thorny evergreen tree or large shrub reaching 8–10 m with a rounded, dense canopy [62,63]. It is a common mid-late successional species in most subtypes of Tamaulipan thornscrub habitats. It fixes atmospheric nitrogen and produces large, edible leguminous fruits [37]. *Ebenopsis* produces fragrant, creamy white, catkin-like flowers from late spring to early fall, with peak flowering from June to August [62]. The flowers and fruit attract many insects, especially nectar-seeking butterflies and bees and a variety of beetles that feed on the seeds and/or their pods [17,63]. *Ebenopsis* serves as a host plant for caterpillars of *Achalarus toxeus* (coyote cloudywing) and *Sphingicampa blanchardi* (Blanchard’s silkmoth) [64]. Mature plants grow slowly, but *Ebenopsis* seedlings have a moderate growth rate, and established individuals are extremely drought-tolerant [63,64]. *Ebenopsis* has a thick seed coat and exhibits morphological dormancy, so mechanical or chemical scarification is required to trigger germination [33]. Treatment with sulfuric acid for 30–35 min has become the standard approach for breaking dormancy and has the added benefit of killing the eggs and larvae of the many seed predators that attack *Ebenopsis* in south Texas [22,31].

*Cordia boissieri* (Mexican olive in English or anacahuita in Spanish; Boraginaceae) is a small, unarmed flowering tree reaching 10 m that is found in most subtypes of Tamaulipan thornscrub habitats and often used as an ornamental [63,64]. It produces large, showy, funnel-shaped white flowers with yellow throats, and blooms year-round but most profusely in late spring to early summer [64,65]. *Cordia* flowers attract butterflies and are pollinated primarily by small beetles and bees, and its fruit is a fleshy, yellow-green, olive-like drupe containing a thick endocarp encasing 2–4 embryos [62,63,64,66]. Birds, deer, and cattle consume the fruits [67], which have medicinal value [62]. Germination of *Cordia* is typically moderate but variable, and often polyembryonic [22]. Seedling survival is significantly lower than other thornscrub species in a nursery setting, but specialized rearing practices have not been explored [22].

*Zanthoxylum fagara* (lime pricklyash in English or colima in Spanish; Rutaceae) is a spreading, rounded shrub or small tree bearing recurved thorns and reaching 5–8 m that occurs as both a canopy and understory species [68]. *Zanthoxylum* produces small, inconspicuous, yellow-green flowers in the spring and numerous small but showy red fruits that ripen in mid to late summer [62,69]. Many birds consume the fruits, deer and many Lepidoptera such as *Papilio cresphontes* (giant swallowtail) and *Eantis tamenund* (northern sicklewing) eat the leaves and young shoots, and many Lepidoptera collect nectar from the small but abundant flowers [62,69,70,71]. A variety of ground-dwelling wildlife use *Zanthoxylum* for shelter because its dense canopy is often thick close to the ground [62]. The flowers, leaves, and fruit have medicinal value and its bark is used as a spice [62,69]. *Zanthoxylum* plants produce a large number of seeds, but propagation from seed has proven difficult, with typically low but variable germination rates [22]. Observed germination rates could be driven by ecological factors impacting seed viability, but, to our knowledge, the effects of common phytohormone treatments on the germination of *Zanthoxylum* seeds have never been studied, and dormancy requirements are uncertain.

### 4.2. Study Site, Experimental Conditions, and Data Collection

The germination portions of this study were conducted in a laboratory at the University of Texas Rio Grande Valley (UTRGV) in Brownville, TX, USA, and the post-germination portions were conducted at the Brownsville Research and Community Garden (BRCG) located outdoors on the UTRGV campus in Brownville, TX, USA (25°53′44.5″ N, 97°28′54.3″ W). Seeds were wild collected at various locations within Cameron and Hidalgo Counties in the summer and fall of 2019, except for one batch of *Cordia* seeds that were wild collected by the USFWS in 2012 and kept in cold storage. *Ebenopsis* and *Zanthoxylum* seeds were primarily collected, with permission, from private residential properties, and the 2019 *Cordia* seeds were collected from the UTRGV Brownsville and Edinburg campuses. *Ebenopsis* seeds were processed by splitting dried pods, separating seeds, and sieving to remove debris. *Zanthoxylum* seeds were separated from harvested branches by hand and sieved to remove debris. *Cordia* seeds were processed within 48 h of collection by first physically stripping the moist flesh from their hard seed coats using coarse sandpaper and rubbing seeds against metal screens with ~13 or ~6 mm openings. *Cordia* seeds were then rinsed in tap water and spread out to dry in a drying oven at 50 °C for one hour prior to storage to prevent molding. All seeds were stored at room temperature (20–22 °C) in a laboratory on the UTRGV Brownsville campus prior to experimental treatments.

The seed treatments described below were performed from October to December 2019. Treated seeds from all treatments were placed into 100 × 15 mm petri dishes on top of a moist paper towel precut to the interior dimensions of the dish, with 20 seeds per dish. Dishes were covered and incubated at 26 °C in a Fisherbrand Isotemp general purpose drying oven (Thermo Fisher Scientific, Waltham, MA, USA). The interior of the opaque incubator was unlit, and variation in temperature was never more than ca. 1 °C, except when the incubator was opened to remove petri dishes for germination surveys, which were performed in a laboratory lit with fluorescent tube bulbs and climate controlled to 20–22 °C. The positions of petri dishes within the incubator were cycled at every survey, and the petri dishes were always moistened with a standard 3 mL of tap water and remoistened on a standard schedule. Painstaking efforts were taken to maintain consistent environmental conditions and to regularly cycle the petri dishes within the incubator, thus it is reasonable to assume that the environmental conditions within the various petri dishes were not significantly different. This approach permitted us to analyze the germination data using classical and generalized linear models that considered each seed as an independent observation. Temperature measurements of different places within the incubator using an infrared thermometer never differed by more than 1.8 °C and were typically less than 1 °C, and all three of the focal species are known to germinate at a considerably broader range of temperatures than those to which they were exposed [22,23,31], which support the validity of our assumption of environmental homogeneity.

Germination data collection began in October 2019 and continued until March 2020. The seeds within each petri dish were surveyed every 48 h for germination or molding for 35 days or until all seeds germinated. Seeds were considered germinated once radicle emergence was distinct. Seeds that were found with mold were discarded. To reduce molding, all seeds were surface sterilized at regular one-week intervals using a 10% bleach solution and gentle agitation for 10 min before being thoroughly rinsed.

Each seedling yielded from the germination portion of our experiments was removed from its petri dish and transplanted into a 3.8 × 3.8 × 20.3 cm (1.5 × 1.5 × 8 in) biodegradable paper container filled with one of the soil mixtures described below. These containers, known as Zipset Plant Bands (Monarch Manufacturing Co., Salida, CO, USA), are the standard type used for reforestation by the USFWS in south Texas. Seedlings were then grown outside at the UTRGV Brownsville Research and Community Garden on greenhouse tables under normal environmental conditions. Seedlings from different treatments were arranged haphazardly and plant bands were placed upright inside shallow, undivided black plastic propagation trays (flats). All treatments were exposed to the same light and watering regimes and were watered approximately every 3 days, unless rainfall occurred. Overall, the growing conditions for the experimental seedlings followed the recommendations of the USFWS for container-grown seedlings used for restoration and did not use any materials or equipment that are not commonly used by commercial growers. That is, growing conditions at the UTRGV nursery were consistent with typical outdoor commercial nursery conditions.

Seedlings were grown in this way from mid October 2019 to March 2020. *Ebenopsis* seedlings were surveyed for survival every two weeks, and the height and leaf count of all live seedlings were quantified three times (approximately every six weeks), including the final data collection in March 2020. During this period, based on weather data collected at the Brownsville-South Padre Island International Airport (station ID USW00012919) located 6.1 km from the study site, the average temperature was 20.6 °C, and average daily high and low temperatures were 26.5 and 15.7 °C, respectively. Temperatures reached as high as 35.6 °C and as low as 2.8 °C. Rainfall in the same period was 134 mm, but this was supplemented with regular watering, and there was no snowfall. Average wind speed was 16.8 km/h with a maximum 2 min wind speed of 62.5 km/h and a maximum 5-s wind speed of 82.1 km/h. No shade or other environmental manipulations were imposed on the experimental seedlings, except when overnight winter conditions were severe enough to threaten seedling survival. On two occasions, seedlings were covered in translucent plastic sheeting when the temperature was forecast to drop below 4 °C or wind speeds were forecast to exceed 40 km/h. Sheeting was removed immediately after severe conditions passed.

### 4.3. Horticultural Treatments

Many horticultural techniques may be employed to break physical or physiological dormancy. This study focuses on three common seed treatments that show promise in promoting germination of thornscrub species but are understudied in this context, as well as the effects of different soil mixes on early seedling growth and survival.

#### 4.3.1. Sulfuric Acid (SA) Treatments

Seeds were coated with sulfuric acid (SA) and either stirred or not stirred (which we refer to as the “stirred” and “soaked” treatments, respectively) for one of five different time intervals. The time intervals differed for each species based on the thickness of their seed coats and were shorter when stirred because the added friction accelerated the chemical degradation of the seed coat. Under a fume hood, seeds were placed in glass beakers and coated with SA by pouring in enough acid to cover all the seeds and then decanting the excess acid. A small amount of sand was added to each stirred treatment to increase friction, and seeds were gently stirred with a glass rod for the prescribed time. *Ebenopsis* seeds were either soaked in SA for 10, 20, 30, 40, 50, or 60 min or stirred in SA for 3, 6, 9, 12, or 15 min; seeds in the SA control treatments were either soaked in water for 30 min or stirred in water for 9 min. *Cordia* seeds were soaked in SA for 20, 40, 60, 80, or 120 min, or soaked in water for 70 min (control). No stirring SA treatments were tested for *Cordia* due to limited seed availability and because we opted to test the effects of heat cracking their seed coats instead (see below). This decision was influenced by preliminary results from the *Ebenopsis* experiments that suggested the longer soaking treatments were both more effective and less labor-intensive. *Zanthoxylum* seeds were either soaked in SA for 1, 2, 3, or 4 min or stirred in SA for 30, 60, 80, or 120 s; seeds in the SA control treatments were soaked in water for 4 min or stirred in water for 120 s. Immediately following SA treatment, all seeds were neutralized in a solution made of 2 parts tap water to 1 part agricultural lime (by volume) for 10 min and then rinsed in tap water. Scarified seeds were then placed on paper towels and observed for approximately 1 h to detect any remaining traces of sulfuric acid. Upon observing no remaining SA, seeds were surfaced sterilized in a 10% bleach solution for 10 min, rinsed thoroughly, and placed in their designated petri dishes as described above.

#### 4.3.2. Gibberellic Acid (GA) Treatments

Seeds of all three focal species were soaked in gibberellic acid (GA or GA_3_) solution for 24 h using one of six concentrations: 0 (control), 5, 10, 50, 100, or 500 mg/L GA. To make the different GA solutions, synthetic gibberellic acid in its solid form (98% purity) was measured out and dissolved in 1–3 mL of ethanol. Dissolved GA was added to 500 mL of water purified via reverse osmosis, followed by 1 mL of Tween Polysorbate 20 (Croda International, Snaith, UK), a surfactant and emulsifier that improves penetration of aqueous solutions into seeds that have limited permeability to water, before adding more water to reach 1000 mL. All solutions were thoroughly mixed prior to use. Seeds were surface sterilized as above and placed into beakers with either 100 mL of GA solution or enough solution to cover the seeds, whichever was greater, and stirred. Seeds were then soaked in their prescribed GA solutions at room temperature for 24 h and stirred 1–2 times in that period. Finally, GA solutions were drained from each beaker, and seeds were immediately placed into their designated petri dishes.

#### 4.3.3. Indole-3-butyric Acid (IBA) Treatments

Seeds of all three focal species subject to IBA treatment were coated in 3% IBA powder (Hormex No. 30; Maia Products, Inc., Westlake Village, CA, USA) immediately prior to being incubated and monitored for germination. Seeds were placed in a petri dish, dusted with IBA powder, and then gently stirred to ensure seeds were totally covered in the powder. Seeds were shaken to remove excess powder and then placed into their designated petri dishes.

#### 4.3.4. *Cordia* Heat Cracking

After stripping the fruit and washing the seeds, all *Cordia* seeds were then oven dried at 50 °C for one hour (to prevent molding), followed by air drying at room temperature for ca. 24 h. However, one batch of *Cordia* seeds, which were collected as fallen fruit at the UTRGV Edinburg campus, were inadvertently left in a drying oven at 50 °C for three days. The hard seed coats of nearly all the *Cordia* seeds in this latter batch had split or cracked while in the drying oven. We were interested in the effect this splitting of the seed coat would have on germination, but we were concerned that a subsequent sulfuric acid treatment would penetrate these gaps and kill the embryos, so we subjected the heat-cracked seeds to their own set of gibberellic acid treatments using the same GA levels as described above.

The heat cracking process may have had other effects related to after-ripening or warm stratification that justify this test. After-ripening occurs when water loss from seeds triggers the conversion of soluble nutrients into their stored forms, which slows or arrests development and can be readily achieved by storing seeds in warm and dry conditions. We did not originally intend to include an after-ripening treatment in this battery of germination trials, but our laboratory accident exposed some *Cordia* seeds to warm and dry conditions, and all of our species were kept in warm and moist conditions following other seed treatments while being monitored for germination.

#### 4.3.5. Soil Mixture Treatments

To examine the effects of different soil characteristics on seedling growth and survival, we subjected each of the *Ebenopsis* seedlings produced in the germination portions of our experiments to one of six different soil mixture treatments as they grew outdoors. All six mixtures were composed of 2–4 components out of five possible types of standard horticultural growing media: peat moss, perlite, sand, topsoil (local origin), and vermiculite. The six soil mixture treatments included: (A) 50% topsoil, 50% vermiculite (the USFWS standard); (B) 50% topsoil, 25% perlite, 25% vermiculite; (C) 50% peat moss, 25% sand, 25% topsoil; (D) 50% peat moss, 25% sand, 25% vermiculite; (E) 50% peat moss, 25% perlite, 25% sand; (F) 50% peat moss, 20% topsoil, 20% vermiculite, 10% perlite. All percentages were based on volume. All soil mixtures also contained Osmocote Pro 19-5-9 slow release granular fertilizer (ICL Fertilizers, Dublin, OH, USA), which was added at the USFWS-recommended rate of 350 mL per 38 L of soil and mixed into the soil until homogenized. The average bulk densities of these soil mixture treatments, measured in g/cm^3^, were A = 0.686, B = 0.675, C = 0.892, D = 0.698, E = 0.702, and F = 0.480.

These five types of growing media were selected primarily based on their availability, affordability, and regular usage by commercial growers. The specific types and grades of growing media utilized in this experiment included: Berger Professional all-purpose medium organic sphagnum peat moss (Berger, Saint-Modeste, QC, Canada), medium-coarse horticultural perlite (KBW Supply, Donna, TX, USA), Sunshine course grade vermiculite (Sun Gro Horticulture, Agawam, MA, USA), natural play sand (Pavestone, Atlanta, GA, USA), and local topsoil provided by the USFWS South Texas Refuge Complex Habitat Restoration Nursery in Alamo, Texas. Local topsoil consists predominantly of Hidalgo sandy clay loam, which is well-drained, moderately permeable, hard, friable, calcareous, and moderately alkaline, taxonomically classified as a hyperthermic Typic Calciustoll [60]. All soil treatments were mixed by adding individual components and the granular fertilizer to a large container and hand blending until a homogenous mixture was observed. The soil mixtures were packed into plant bands by hand and compressed by dropping the bands onto a hard surface until soil did not flow out when the bands were upright.

#### 4.3.6. Soil Warming Using Heating Mats

To examine the effects of soil temperature on seedling growth and survival, we subjected half of the *Ebenopsis* seedlings produced in the germination portions of our experiments to a soil warming treatment using electric heating mats as seedlings grew outdoors. As seeds germinated and were planted, seedlings in the same soil treatment were alternately assigned to either the soil warming treatment or the control and were placed outdoors on a greenhouse table either on top of a heating mat set at 36 °C or without a heating mat, respectively. Heating mats are often used when rearing young seedlings during the winter to promote seedling growth and root development, but their effects on thornscrub species if employed during the early spring were unclear.

### 4.4. Root Morphology Analyses

The oldest *Ebenopsis* seedlings, aged predominantly 90–110 days, were harvested for root analysis after the final seedling survey. Ten samples from each soil treatment (evenly split between the two soil warming treatments) were randomly selected from those *Ebenopsis* seedlings subjected to SA treatment for 50 or 60 min without stirring. We used seedlings from only two seed treatments because root morphological analyses are time consuming and available machine time was limited, so our sample size was limited, and we wanted to maximize our power to detect differences between the soil type and soil warming treatments and their interactions. We used the 50 and 60 min SA treatments because their high germination rates and low times to germination meant they were most common among the oldest available seedlings, and because the oldest seedlings were likely to have the most developed root systems. Stems were cut at the soil surface, plant bands were cut vertically along one corner, and the root ball was removed. Root samples were then gently rinsed by pouring water over samples to wash away the soil around and on the roots until all soil was removed. Root samples were then placed in plastic bags, refrigerated prior to measurement, and measured within 24 h.

Using a WinRHIZO scanner and associated software (Regent Instruments Inc., Québec, QC, Canada), we quantified the root length, root surface area, average root diameter, root volume, and the abundances of root tips, root forks, and root crossings for each root sample. Root samples were placed in transparent trays and floated in water prior to and during scanning to maintain their three-dimensional structure. Preset parameters for tray size were used, and the WinRHIZO software auto-quantified our root morphology response variables based on the scans.

### 4.5. Statistical Analyses

For each of our binary response variables (seed germination, seed molding, and seedling survival), we examined differences among experimental treatments by fitting a generalized linear model (GLM) for each applicable species using the ‘glm’ function in R version 4.0.5 with a binomial distribution family and model terms for any applicable treatments (R Foundation for Statistical Computing, Vienna, Austria). For each GLM, we performed an analysis of deviance (ANODEV) for hypothesis testing followed by a least square means post-hoc test (‘lsmeans’ function in R), where applicable, to identify any significant differences between treatment levels. For each of our continuous response variables (time to germination, time to seed molding, seedling height, seedling leaf count, root length, root surface area, average root diameter, root volume, and the abundances of root tips, root forks, and root crossings), we fit linear models using the ‘lm’ function in R with model terms for applicable treatments. We then used analysis of variance (ANOVA) or, if a model included seedling age, analysis of covariance (ANCOVA) to evaluate the effects of our experimental treatments with least square means post-hoc tests to compare treatment levels.

Prior to analyses, because the ages of seedlings varied at the time of measurement, seedling height, leaf count, and all the root metrics were normalized by dividing these values by the observed seedling’s age in days. We performed Shapiro–Wilk tests of normality (‘shapiro.test’ function in R) on model residuals and Breusch–Pagan tests for homoscedasticity (‘bptest’ function in R) to assess whether our models met the assumptions of ANOVA. Accordingly, we log transformed the time to germination for *Ebenopsis* treated with sulfuric acid, time to germination for *Cordia* treated with gibberellic acid, and the abundance of *Ebenopsis* root tips, and we square root transformed the average root diameter of *Ebenopsis* to better conform to the assumptions of ANOVA. Where appropriate, either because of a relatively low number of observations or to conform to the assumptions of ANOVA, we performed stepwise model pruning using the ‘step’ function in R to simplify models by removing terms that were not significant and explained the least observed variance. We used Tukey-adjustments in our least square means post-hoc tests when comparing more than 12 treatment levels, otherwise our post-hoc tests were unadjusted [72]. In all cases, a probability value of *p* < 0.05 was used to determine significance

## 5. Conclusions

Sulfuric acid (SA) treatment of *Ebenopsis* seeds for 40–60 min proved effective in promoting germination without diminishing seedling survival or growth. Gibberellic acid (GA) treatment alone did not promote *Ebenopsis* germination, but its effects in combination with SA treatment merits study. Different soil mixture types influenced seedling survival and growth, but most patterns were subtle, and the top performing soil type was the standard 50% topsoil, 50% vermiculite mix (plus time-release granular fertilizer) used and recommended by USFWS. Heating generally did not improve seedling growth, even during cooler months, but some benefits of heating were observed in soil type A.

Treating *Cordia* seeds with 100 mg/L GA solution may improve germination, and so might heat cracking the endocarp of *Cordia* seeds using the new technique we describe. Seed viability of both *Cordia* and *Zanthoxylum* was very low in other germination trials. IBA powder did not promote germination of any of our focal species, but its efficacy remains uncertain because the viability of the seeds tested were unknown. Viability of seeds should always be assessed using tetrazolium or a similar approach to both provide critical baseline data for thornscrub species and to minimize uncertainty when experimenting with methods to break dormancy and/or improve germination.

Several avenues of future research were identified in the discussion, but most focus on evaluating the combined effects of multiple seed treatments or identifying the drivers of variability in seed viability or germination. Our results suggest that soil microorganisms may have both significantly influenced seedling performance and been influenced themselves by our horticultural treatments; this merits future study. Advances in propagating thornscrub species beyond what is possible from standard horticultural approaches may be yielded from exploration of the understudied ecological relationships and plant–animal interactions associated with pollination and seed dispersal in thornscrub habitats. For example, microorganisms associated with nurse plants or gut passage in key dispersers may overcome troublesome barriers to germination or seedling survival. Alternatively, seed–herbivore interactions may be key to overcoming seed limitation or low viability. Some of this information will be more immediately relevant to commercial nursery growers than others, but all of these research directions will help fill the currently large and abundant knowledge gaps in the propagation of Tamaulipan thornscrub species and the restoration of their highly valuable habitats.

## Figures and Tables

**Table 1:** Summary of results for chemical seed treatments for all three focal species. Germination likelihood (%), average time to germination (days), and molding likelihood (%) are shown for each species, treatment type, and dosage within each treatment type.

Treatment		*Ebenopsis ebano*				*Cordia boissieri*				*Zanthoxylum fagara*			
		Dose	Germ.	Days to Germ.	Mold	Dose	Germ.	Days to Germ.	Mold	Dose	Germ.	Days to Germ.	Mold
Sulfuric acid (SA)	Stirred	Control	4%	22	0%					Control	0%	-	0%
		3 min	4%	1	4%					30 s	0%	-	0%
		6 min	24%	10.2	0%					60 s	0%	-	0%
		9 min	68%	14.8	0%					80 s	4%	10	0%
		12 min	60%	11.2	0%					120 s	0%	-	0%
		15 min	92%	11.2	0%								
	Not stirred	Control	0%	-	8%	Control	0%	-	5%	Control	0%	-	0%
		10 min	0%	-	8%	20 min	0%	-	0%	1 min	0%	-	0%
		20 min	60%	9.3	8%	40 min	0%	-	3%	2 min	0%	-	0%
		30 min	84%	7.9	8%	60 min	0%	-	0%	3 min	0%	-	0%
		40 min	92%	7.5	0%	80 min	0%	-	0%	4 min	0%	-	0%
		50 min	100%	7.6	0%	120 min	3%	17	5%				
		60 min	92%	7.9	0%								
Gibberellic acid (GA)	Cracked					Control	30%	6.5	0%				
						5 mg/L	48%	10.7	0%				
						10 mg/L	30%	9.5	0%				
						50 mg/L	35%	8.9	0%				
						100 mg/L	63%	12.1	0%				
						500 mg/L	35%	7.8	0%				
	Not cracked	Control	0%	-	0%	Control	0%	-	0%	Control	0%	-	0%
		5 mg/L	5%	9	0%	5 mg/L	0%	-	0%	5 mg/L	0%	-	0%
		10 mg/L	10%	26.5	0%	10 mg/L	3%	11	0%	10 mg/L	0%	-	0%
		50 mg/L	3%	21	3%	50 mg/L	13%	10.2	0%	50 mg/L	0%	-	0%
		100 mg/L	15%	18	5%	100 mg/L	17%	8.9	0%	100 mg/L	0%	-	0%
		500 mg/L	10%	27	5%	500 mg/L	20%	11.8	0%	500 mg/L	0%	-	0%
Indole-3-butyric acid (IBA)		Control	0%	-	3%	Control	0%	-	0%	Control	0%	-	0%
		3% pwdr	0%	-	8%	3% pwdr	0%	-	5%	3% pwdr	0%	-	0%

**Table 2:** ANODEV results examining the effects of gibberellic acid (GA) concentration, seed coat heat cracking, and their interaction on germination likelihood of *Cordia boissieri* seeds. Legend: **, 0.001 ≤ *p* < 0.01; *** *p* < 0.001.

Factor	d.f.	χ^2^	*p*	
Gibberellic acid concentration	5	17.30	0.0040	**
Seed coat heat cracking	1	70.57	<0.0001	***
GA conc. × seed coat	5	19.04	0.0019	**
Model	11	106.92	<0.0001	***

**Table 3:** ANOVA results examining the effects of gibberellic acid (GA) concentration, seed coat heat cracking, and their interaction on time to germination of *Cordia boissieri* seeds.

Factor	d.f.	F_9,107_	*p*
Gibberellic acid conc.	5	1.47	0.20
Seed coat heat cracking	1	0.16	0.70
GA conc. × seed coat	3	1.44	0.24
Model	9	1.31	0.24

**Table 4:** ANODEV results examining the effects of sulfuric acid (SA) treatment, soil mixture type, soil warming via heating mat, and the interaction of soil type × warming on the survival of *Ebenopsis ebano* seedlings. Legend: *, 0.01 ≤ *p* < 0.05; **, 0.001 ≤ *p* < 0.01.

Factor	d.f.	χ^2^	*p*	
SA treatment	11	13.40	0.27	
Soil type	5	12.70	0.0266	*
Warming	1	8.23	0.0041	**
Soil × warming	5	3.01	0.70	
Model	22	37.31	0.0219	*

**Table 5:** ANCOVA results examining the effects of seedling age, soil mixture type, soil warming via heating mat, and the interactions of age × warming and soil type × warming on the height of *Ebenopsis ebano* seedlings. For this model and all others examining *Ebenopsis* seedling performance, we initially considered a full model with terms for seedling age, sulfuric acid (SA) treatment, soil type, warming, and all possible interactions, and we then pruned the model. Legend: ., 0.05 ≤ *p* < 0.1; *, 0.01 ≤ *p* < 0.05; ***, *p* < 0.001.

Factor	d.f.	F_13,199_	*p*	
Age	1	62.25	<0.0001	***
Soil type	5	2.09	0.0683	.
Warming	1	3.43	0.0656	.
Age × warming	1	3.85	0.0512	.
Soil type × warming	5	2.85	0.0165	*
Model	13	7.00	<0.0001	***

**Table 6:** ANCOVA results examining the effects of seedling age, sulfuric acid (SA) treatment, soil mixture type, soil warming, and the interactions of age × soil type and soil type × warming on the leaf abundance of *Ebenopsis ebano* seedlings. Legend: *, 0.01 ≤ *p* < 0.05; **, 0.001 ≤ *p* < 0.01; *** *p* < 0.001.

Factor	d.f.	F_28,184_	*p*	
Age	1	117.27	<0.0001	***
SA treatment	11	2.11	0.0218	*
Soil type	5	5.37	0.0001	***
Warming	1	1.62	0.20	
Age × soil type	5	6.12	<0.0001	***
Soil type × warming	5	4.24	0.0011	**
Model	28	10.75	<0.0001	***

**Table 7:** ANCOVA results examining the effects of seedling age, sulfuric acid (SA) treatment, soil mixture type, soil warming, and the interactions of age × soil type, age × warming, SA treatment × warming, and soil type × warming on the root length of *Ebenopsis ebano* seedlings. Legend: *, 0.01 ≤ *p* < 0.05.

Factor	d.f.	F_22,37_	*p*	
Age	1	5.81	0.0210	*
SA treatment	2	4.89	0.0131	*
Soil type	5	3.51	0.0108	*
Warming	1	5.22	0.0282	*
Age × soil type	5	3.50	0.0108	*
Age × warming	1	5.10	0.0299	*
SA × warming	2	1.69	0.20	
Soil type × warming	5	1.98	0.10	
Model	22	2.26	0.0141	*

**Table 8:** ANCOVA results examining the effects of seedling age, sulfuric acid (SA) treatment, soil mixture type, soil warming, and the interactions of age × soil type, age × warming, SA treatment × warming, and soil type × warming on the root surface area of *Ebenopsis ebano* seedlings. Legend: ., 0.05 ≤ *p* < 0.1; *, 0.01 ≤ *p* < 0.05; **, 0.001 ≤ *p* < 0.01.

Factor	d.f.	F_22,37_	*p*	
Age	1	6.78	0.0132	*
SA treatment	2	5.41	0.0087	**
Soil type	5	2.18	0.0775	.
Warming	1	4.99	0.0316	*
Age × soil type	5	2.13	0.0833	.
Age × warming	1	5.02	0.0312	*
SA × warming	2	1.98	0.15	
Soil type × warming	5	1.44	0.23	
Model	22	1.86	0.0470	*

**Table 9:** ANCOVA results examining the effects of seedling age, sulfuric acid (SA) treatment, soil mixture type, soil warming, and the interactions of age × soil type, age × warming, and SA soil type × warming on the average root diameter of *Ebenopsis ebano* seedlings. Legend: ., 0.05 ≤ *p* < 0.1; *, 0.01 ≤ *p* < 0.05; **, 0.001 ≤ *p* < 0.01.

Factor	d.f.	F_20,39_	*p*	
Age	1	4.01	0.0523	.
SA treatment	2	4.14	0.0235	*
Soil type	5	3.76	0.0071	**
Warming	1	4.87	0.0333	*
Age × soil type	5	3.83	0.0064	**
Age × warming	1	4.45	0.0414	*
Soil type × warming	2	2.29	0.0642	.
Model	20	2.70	0.0039	**

**Table 10:** ANCOVA results examining the effects of seedling age, sulfuric acid (SA) treatment, soil mixture type, soil warming, and the interactions of age × SA treatment and age × soil type on the root volume of *Ebenopsis ebano* seedlings. Legend: *, 0.01 ≤ *p* < 0.05; **, 0.001 ≤ *p* < 0.01.

Factor	d.f.	F_16,43_	*p*	
Age	1	2.08	0.16	
SA treatment	2	1.33	0.28	
Soil type	5	3.35	0.0121	*
Warming	1	5.30	0.0262	*
Age × SA	2	1.35	0.27	
Age × soil type	5	3.63	0.0079	**
Model	16	2.93	0.0026	**

**Table 11:** ANCOVA results examining the effects of seedling age, soil mixture type, soil warming, and the interactions of age × soil type and soil type × warming on root tip abundance of *Ebenopsis ebano* seedlings. Legend: *, 0.01 ≤ *p* < 0.05; *** *p* < 0.001.

Factor	d.f.	F_17,40_	*p*	
Age	1	1.76	0.19	
Soil type	5	12.36	<0.0001	***
Warming	1	5.84	0.0203	*
Age × soil type	5	3.19	0.0163	*
Soil type × warming	5	2.63	0.0381	*
Model	17	7.48	<0.0001	***

**Table 12:** ANCOVA results examining the effects of seedling age, SA treatment, soil type, soil warming, and the interactions of age × soil type, age × warming, and SA treatment × warming on root fork abundance of *Ebenopsis ebano* seedlings. Legend: *, 0.01 ≤ *p* < 0.05; **, 0.001 ≤ *p* < 0.01; *** *p* < 0.001.

Factor	d.f.	F_17,41_	*p*	
Age	1	8.40	0.0060	**
SA treatment	2	9.27	0.0005	***
Soil type	5	7.75	<0.0001	***
Warming	1	12.11	0.0012	**
Age × soil type	5	7.68	<0.0001	***
Age × warming	1	11.87	0.0013	**
SA × warming	2	4.78	0.0137	*
Model	17	3.66	0.0003	***

**Table 13:** ANCOVA results examining the effects of seedling age, sulfuric acid (SA) treatment, soil mixture type, soil warming, and the interactions of age × soil type, SA treatment × soil type, and SA treatment × warming on root crossing abundance of *Ebenopsis ebano* seedlings. Legend: ., 0.05 ≤ *p* < 0.1; *, 0.01 ≤ *p* < 0.05; **, 0.001 ≤ *p* < 0.01.

Factor	d.f.	F_32,27_	*p*	
Age	1	9.18	0.0053	**
SA treatment	2	4.31	0.0238	*
Soil type	5	2.16	0.0884	.
Warming	1	7.57	0.0105	*
Age × soil type	5	2.08	0.0989	.
Age × warming	1	7.54	0.0106	*
SA × soil type	10	1.16	0.36	
SA × warming	2	3.47	0.0456	*
Soil type × warming	5	2.01	0.11	
Model	32	2.75	0.0045	**

**Table 14:** Summary of relative performance of *Ebenopsis ebano* seedlings in each soil type as compared to other soil types. Legend: +, higher; =, intermediate; −, lower. See Table 5, Table 6, Table 7, Table 8, Table 9, Table 10, Table 11, Table 12 and Table 13 and Figure 6, Figure 7, Figure 8, Figure 9, Figure 10, Figure 11, Figure 12, Figure 13 and Figure 14 for greater detail.

Response Variable or Other Factor	*p*-Value (Soil Type)	Soil Mixture Type					
		A	B	C	D	E	F
Height	0.0683	+	+	+	+	=	−
Leaf abundance	0.0001	=	+	−	−	=	+
Root length	0.0108	−	−	=	=	+	=
Root surface area	0.0775	=	−	+	=	=	=
Root average diameter	0.0071	+	=	=	−	=	=
Root volume	0.0121	+	=	+	−	−	=
Root tips	<0.0001	−	−	=	=	+	+
Root forks	<0.0001	+	−	=	=	=	=
Root crossings	0.0884	+	−	+	+	+	−
Water-holding capacity	Qualitative	=	−	=	+	=	+
Bulk density	Direct measure	=	=	+	=	=	−

**Table 15:** Nature of the linear relationships between *Ebenopsis ebano* seedling age and performance metrics for each soil type. Legend: ++, strongly positive; +, positive; =, neutral and/or not significant; −, negative; −−, strongly negative. See Table 5, Table 6, Table 7, Table 8, Table 9, Table 10, Table 11, Table 12 and Table 13 and Figure 6, Figure 7, Figure 8, Figure 9, Figure 10, Figure 11, Figure 12, Figure 13 and Figure 14 for greater detail.

Response Variable	*p*-Value (Age × Soil Type)	Soil Mixture Type					
		A	B	C	D	E	F
Height	> 0.1	n.s.	n.s.	n.s.	n.s.	n.s.	n.s.
Leaf abundance	<0.0001	+	++	+	+	++	++
Root length	0.0108	−−	+	−	+	+	=
Root surface area	0.0833	−	+	=	=	+	=
Root average diameter	0.0064	++	−	+	−	−	−
Root volume	0.0079	+	+	++	−	−	−
Root tips	0.0163	−	=	−	=	+	+
Root forks	<0.0001	−	+	=	+	+	=
Root crossings	0.0989	−−	+	−−	+	+	−

**Table 16:** Comparisons of *Ebenopsis ebano* seedling performance metrics in the soil warming treatment compared to the unheated control for each soil type. Legend: ++, significant increase; +, notable but not significant increase; =, negligible difference; −, notable but not significant decrease; −−, significant decrease. See Table 5, Table 6, Table 7, Table 8, Table 9, Table 10, Table 11, Table 12 and Table 13 and Figure 6, Figure 7, Figure 8, Figure 9, Figure 10, Figure 11, Figure 12, Figure 13 and Figure 14 for greater detail.

Response Variable	*p*-Value (Soil Type × Warming)	Soil Mixture Type					
		A	B	C	D	E	F
Height	0.0165	++	=	=	=	−−	−
Leaf abundance	0.0011	+	=	=	=	−−	−
Root length	>0.1	n.s.	n.s.	n.s.	n.s.	n.s.	n.s.
Root surface area	>0.1	n.s.	n.s.	n.s.	n.s.	n.s.	n.s.
Root average diameter	0.0642	−−	=	+	=	−	−−
Root volume	>0.1	n.s.	n.s.	n.s.	n.s.	n.s.	n.s.
Root tips	0.0381	=	−	=	−−	−	=
Root forks	>0.1	n.s.	n.s.	n.s.	n.s.	n.s.	n.s.
Root crossings	>0.1	n.s.	n.s.	n.s.	n.s.	n.s.	n.s.

## Data Availability

The data generated by this experiment and used for the analyses reported herein are available upon request from the corresponding author.
